# Dual-Pronged Lipid Nanocarriers Promote Immunotherapy for TNBC by Inducing Immunogenic Cell Death and Activating Lymphoid Immune Cells

**DOI:** 10.34133/research.1139

**Published:** 2026-04-13

**Authors:** Jun Ye, Shiyuan Wang, Siyi Wu, Renjie Li, Xiang Li, Shan Chen, Yujie Wang, Lu Wang, Caiyun Qin, Hongliang Wang, Yanfang Yang, Jiani Wang, Tingting Du, Fei Ma, Yuling Liu

**Affiliations:** ^1^ State Key Laboratory of Bioactive Substance and Function of Natural Medicines, Institute of Materia Medica, Chinese Academy of Medical Sciences & Peking Union Medical College, Beijing 100050, China.; ^2^ Beijing Key Laboratory of Key Technologies for Natural Drug Delivery and Novel Formulations, Institute of Materia Medica, Chinese Academy of Medical Sciences & Peking Union Medical College, Beijing 100050, China.; ^3^ Department of Pharmacy, China-Japan Friendship Hospital, Beijing 100029, China.; ^4^ Beijing Wehand-Bio Pharmaceutical Co., Ltd., Beijing 100260, China.; ^5^ National Cancer Center/National Clinical Research Center for Cancer/Cancer Hospital, Chinese Academy of Medical Sciences & Peking Union Medical College, Beijing 100021, China.

## Abstract

Triple-negative breast cancer (TNBC) is recognized as the notoriously difficult molecular subtype of breast cancer to manage therapeutically, creating a pressing need for more effective treatment approaches. Immunochemotherapy is an emerging strategy with great clinical potential, but most focus on the tumor microenvironment itself and ignore the immune regulation of lymph nodes. Herewith, we report a dual-pronged approach of tumor-targeted paclitaxel-encapsulated nanoemulsion (PTX Emul, Phase II clinical trial) and lymph node-targeted chlorogenic acid-encapsulated self-emulsifying nanocarriers (CHA-SME) to elicit strong and selective immunogenic cell death (ICD) in the tumor and to activate immune cells in the lymph nodes, respectively, to achieve highly effective cancer immunotherapy. PTX Emul exhibited evident tumor targetability and superior tumor accumulation, induced potent ICD, and then efficiently stimulated the maturation of dendritic cells (DCs). CHA-SME demonstrates a substantial capacity to enhance drug accumulation within the mesenteric lymph nodes through the lymphatic transport pathway. Of note, PTX Emul combined with CHA-SME augmented the immunotherapeutic effects through highly efficient ICD induction within the tumor microenvironment of 4T1 orthotopic tumor, the potent DC maturation, and the effective activation of T cell-based antitumor immunity, resulting in a substantial enhancement in the inhibition of 4T1 orthotopic tumors and, notably, a reduction in lung metastasis. The dual-pronged approach of TNBC-targeted PTX Emul and lymph node-targeted CHA-SME by inducing ICD and activating lymphoid immune cells, which amplifies the systemic antitumor immune response, provides an interesting platform for potent immunochemotherapy of TNBC.

## Introduction

Among breast cancer subtypes, triple-negative breast cancer (TNBC) is characterized as the most therapeutically challenging. Its unique molecular profile presents complex therapeutic challenges, resulting in limited therapeutic options, the worst prognosis, and the highest mortality rates. TNBC exhibits resistance to both endocrine and targeted therapies, rendering chemotherapy the primary therapeutic approach [1–3]. Immune checkpoint blockade shows promise for treating TNBC; however, clinical studies have reported only modest responses, leading to investigations of its combination with chemotherapy [4–9]. The paradigm of combining immunotherapy with chemotherapy is currently the focus of research and clinical trials [7,10–12]. This reliance on chemotherapy is not solely due to its current status as the primary treatment modality for TNBC, but also because a subset of chemotherapeutic drugs, including paclitaxel, oxaliplatin, and mitoxantrone, have been demonstrated to enhance immunogenicity within the tumor microenvironment. These agents synergistically modulate the immune response by inducing immunogenic cell death (ICD) [13–15]. The process of ICD involves the exposure and secretion of damage-associated molecular patterns (DAMPs) like calreticulin (CRT), high-mobility group box 1 (HMGB1), and adenosine triphosphate (ATP) from tumor cells. These molecules recruit dendritic cells (DCs) to process tumor antigens, thereby promoting T cell activation and facilitating the adaptive immune response [16].

However, it is challenging to obtain the desired antitumor immune effect by relying solely on the ICD within the tumor microenvironment. In the whole process of an immune response, in addition to the tumor microenvironment, the lymph nodes, as one of the key immune organs, are not only the core sites for regulating and maintaining the immune response but also an important organ for lymphatic metastasis in the early stage of TNBC [17–19]. However, the immune cells in the lymph nodes usually show a state of tolerance under the influence of cytokines secreted by the tumor microenvironment [20]. Therefore, in response to the dual dilemma of the immunosuppressive microenvironment and immune tolerance within the lymph nodes, the combination of chemotherapy with immunotherapy to achieve a dual-pronged therapeutic paradigm against TNBC is promising for clinical application. Chemotherapy induces ICD in tumor cells to increase immunogenicity within the tumor microenvironment, and immunotherapy activates immune cells within lymph nodes to overcome immune cell tolerance.

In this study, chemotherapeutic drug paclitaxel-encapsulated lipid nanoemulsion (PTX Emul) and immunomodulatory agent chlorogenic acid-encapsulated self-microemulsifying nanocarriers (CHA-SME) were used in a dual-pronged approach for the treatment of TNBC. PTX Emul possesses a core–shell architecture of 160 nm, wherein the core comprises an oil phase containing dissolved PTX and the shell consists of a single layer of phospholipids [21]. A preliminary study confirms enhanced antitumor activity and improved safety in comparison to PTX injection against TNBC, which may be attributed to the active targeting pathway mediated by low-density lipoprotein (LDL) receptors [22,23]. PTX Emul is in the process of a Phase II clinical trial (ClinicalTrials.gov, ID: NCT06513364) to treat breast cancer. The tumor-targeting profile of PTX Emul contributes to its ability to induce potent ICD effects within the tumor microenvironment. Chlorogenic acid (CHA), a compound prevalent in Chinese herbal medicine, exhibits a range of beneficial properties, notably its antitumor activity, which is facilitated through immunomodulatory pathways [24]. To enhance the immunomodulatory effect of CHA, CHA-SME (~60 nm) was engineered to facilitate targeted delivery to mesenteric lymph nodes (MLNs). This CHA-SME formulation substantially enhances drug accumulation in the MLNs via the lymphatic transport route. Furthermore, it effectively primes naive T cells into effector T cells, thereby suppressing glioma tumor growth [25,26]. CHA-SME is expected to improve the tolerance status of immune cells due to its excellent lymphatic targeting and immune activation effects, and further enhance the immunotherapeutic effects when combined with PTX Emul.

**Fig. 1.**
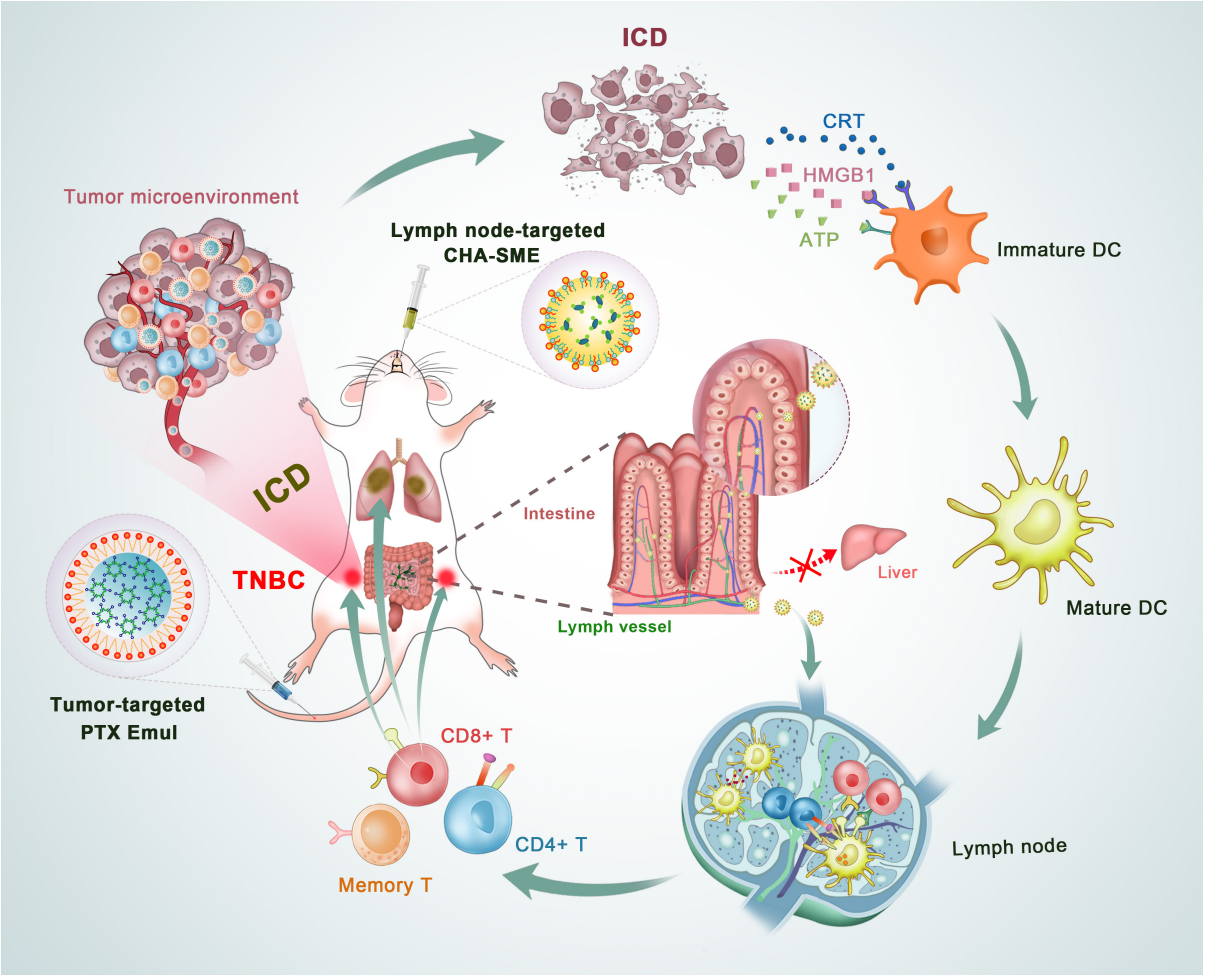
Illustration of the dual-pronged approach of TNBC-targeted PTX Emul and lymph node-targeted CHA-SME to elicit potent ICD in the tumor and to activate immune cells within the lymph nodes, respectively, to achieve highly effective cancer immunotherapy for TNBC.

Given the key roles of the tumor microenvironment and lymph nodes in the tumor immune response, we report a dual-pronged approach of TNBC-targeted PTX Emul and lymph node-targeted CHA-SME to elicit potent ICD within the tumor microenvironment and to activate immune cells within the lymph nodes, respectively, to achieve highly effective cancer immunotherapy for TNBC (Fig. 1). The TNBC-targeted PTX Emul could specifically target the LDL receptors expressed on the 4T1 cells for efficient drug delivery to tumors, where the sustained release of PTX from the nanoemulsion could induce robust ICD in tumor cells and release damage-associated molecules to promote DC activation. Synchronously, the lymph node-targeted CHA-SME could efficiently accumulate in the MLNs through the lymphatic transport pathway, where the gradual release of CHA could precisely regulate the function of DCs and T lymphocytes in the lymphatic nodes, improve immune tolerance, and synergize with the ICD effect to boost the body’s antitumor immune response systemically. This study demonstrates that a dual-pronged approach focusing on dual immunomodulation within the tumor microenvironment and lymph nodes with 2 rationally designed targeted lipid nanocarriers can greatly improve the antitumor immune response, presenting a promising approach for the effective immunotherapy of TNBC.

## Results and Discussion

### Characteristics of PTX Emul and CHA-SME

**Fig. 2.**
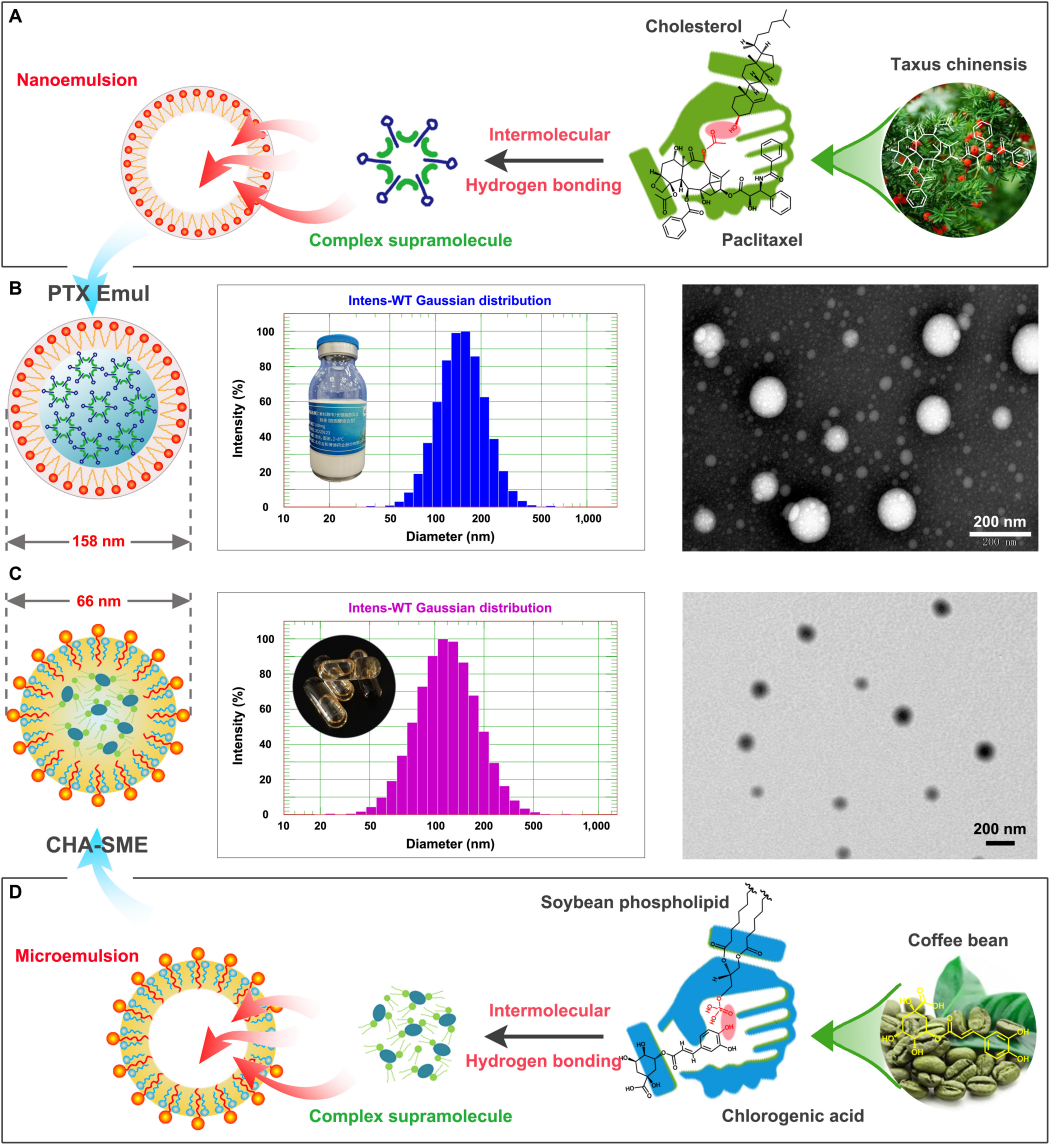
Properties and characteristics of PTX Emul and CHA-SME. (A) Diagrammatic representation of the PTX Emul preparation workflow. (B) The schematic structure, particle size distribution, and morphology of PTX Emul. Scale bar = 200 nm. (C) The schematic structure, particle size distribution, and morphology of CHA-SME. Scale bar = 200 nm. (D) Schematic diagram of the preparation workflow of CHA-SME.

Lipid nanoemulsions have attracted considerable interest in drug delivery due to their exceptional properties, including nanoscale dimensions, extensive surface area, high drug encapsulation efficiency, enhanced stability, and capability for targeted drug delivery [27,28]. The established clinical history of lipid emulsion products (e.g., Intralipid, Liple, and Diprivan) since the 1960s affirms their safety profile and translational potential for pharmacological delivery applications [29]. Lipid emulsions (oil-in-water) typically have a core–shell architecture, with an oil core acting as a storage site for lipophilic drugs, while the phospholipid monolayer shell is essential for nanoparticle stabilization. Our prior study engineered a PTX-encapsulated, tumor-targeting lipid nanoemulsion (PTX Emul) of ideal nanoscale dimensions. Relative to PTX injection, PTX Emul showed enhanced tumor-targeting specificity, more efficient tumor accumulation, and superior in vivo antitumor activity in breast cancer [21–23,30,31]. Based on its superior tumor-targeting ability and antitumor activity, PTX Emul was approved by the National Medical Products Administration for clinical study in 2019 and is currently undergoing a Phase II clinical trial (ClinicalTrials.gov, ID: NCT06513364). In this study, PTX Emul, based on the PTX–cholesterol complex and produced by high-pressure homogenization (Fig. 2A), is well dispersed and presents a homogeneous milky white suspension. The average hydrodynamic diameter of PTX Emul is ~158 nm with a polydispersity index (PDI) of 0.127, indicating a relatively uniform particle size distribution. PTX Emul exhibits a spherical-like structure with a homogeneous size under transmission electron microscopy (TEM) (Fig. 2B).

Self-microemulsifying nanocarriers (SME) are uniform blends of oils, surfactants, solvents, and co-solvents that automatically create fine oil-in-water emulsions upon aqueous dilution [32]. SME have emerged as an attractive oral delivery vehicle for their capacity to direct drugs into the intestinal lymphatic system. This transport route offers a key pharmacological advantage: it circumvents the hepatic first-pass effect and promotes drug residence in MLNs, leading to substantially improved bioavailability and therapeutic efficacy [33–35]. Thus, an appealing potential advantage of SME in the context of lymphatic transport is their capability to assist in targeting immunomodulators to the MLNs, thereby facilitating the direct action of immunomodulators on immune cells. In this study, CHA-SME was engineered for targeted delivery of the immunomodulator CHA to MLNs to precisely modulate the function of immune cells therein to exert tumor immune-enhancing effects. The particle size distribution and morphology of CHA-SME were analyzed by DLS and TEM, respectively. As shown in Fig. 2C and D, the average hydrodynamic diameter of the spherical CHA-SME was about 66 nm, with a PDI measured at 0.197.

Importantly, both nanocarrier platforms were selected based on their established clinical translation pathways, which considerably de-risks the translational potential of this combination strategy. The PTX Emul, built upon the clinically validated lipid nanoemulsion technology, is already in Phase II clinical evaluation for breast cancer (NCT06513364), confirming its manufacturability, stability, and preliminary safety profile in humans. Similarly, CHA-SME is based on the self-microemulsifying drug delivery system, a widely adopted platform for oral delivery with multiple approved products, facilitating its path toward clinical application. This strategic use of clinically grounded delivery systems enhances the feasibility of rapidly translating this dual-targeting immunochemotherapy strategy into clinical evaluation for TNBC patients.

Given the considerable benefits of chemotherapy in combination with immunotherapy, we report the dual-pronged approach of TNBC-targeted PTX Emul (~158 nm) and lymph node-targeted CHA-SME (~66 nm) to elicit potent ICD within the tumor microenvironment and to activate lymphocytes within the MLNs, respectively, to achieve highly effective cancer immunotherapy for TNBC.

### LDLR-mediated cellular uptake profile of PTX Emul

**Fig. 3.**
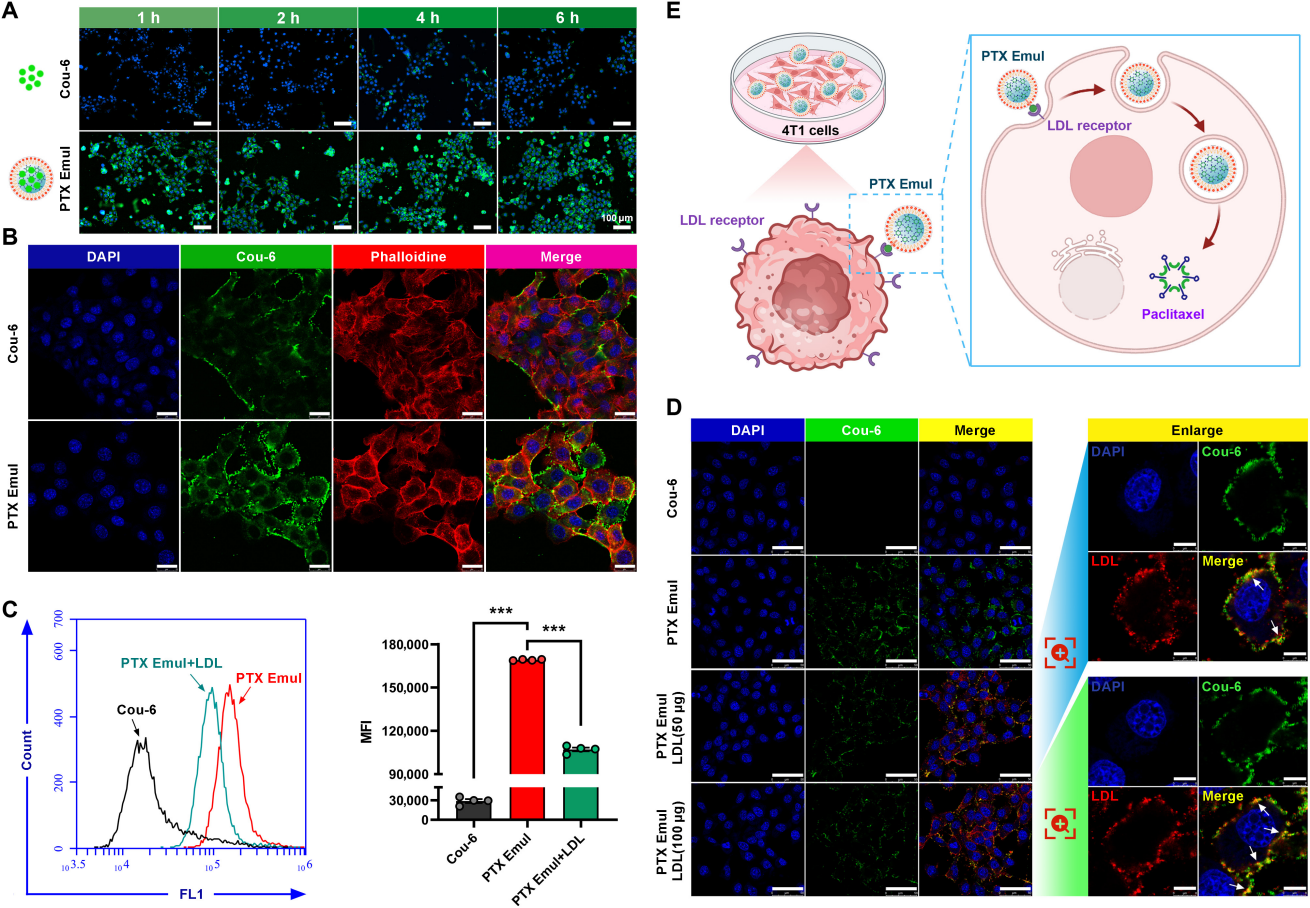
In vitro analysis of cellular internalization of PTX Emul labeled with Cou-6 in 4T1 cells. (A) Representative fluorescence photographs of 4T1 cells after incubation with Cou-6 or Cou-6-labeled PTX Emul (green) for 1, 2, 4, and 6 h. Scale bar = 100 μm. (B) Representative CLSM images of 4T1 cells after incubation with Cou-6 or Cou-6-labeled PTX Emul (green) for 2 h. Scale bar = 25 μm. (C) The analysis of 4T1 cells using flow cytometry involved incubation with Cou-6, Cou-6-labeled PTX Emul, or Cou-6-labeled PTX Emul along with LDL. All data are expressed as mean ± SEM (*n* = 4). ***P < 0.001. (D) Representative CLSM photographs of 4T1 cells after incubation with Cou-6, Cou-6-labeled PTX Emul, Cou-6-labeled PTX Emul plus 50 μg of DiI-labeled LDL, or Cou-6-labeled PTX Emul (green) plus 100 μg of DiI-labeled LDL (red). Scale bars = 50 μm (left panel) and 8 μm (right panel), respectively. (E) Diagram illustrating the efficient internalization of PTX Emul by 4T1 cells via an LDL receptor-mediated active targeting mechanism.

The 4T1 mouse TNBC cell line served as an in vitro model to explore the targeted uptake profile of PTX Emul using confocal laser scanning microscopy (CLSM) for qualitative analysis and flow cytometry for quantitative analysis. To trace the intracellular transport fate of PTX Emul, PTX Emul was labeled with the green fluorescent dye Cou-6 (Cou-6 labeled PTX Emul). The green fluorescence intensity of Cou-6-labeled PTX Emul in 4T1 cells rose progressively with incubation times ranging from 1 to 6 h (Fig. 3A), suggesting that PTX Emul is taken up by 4T1 cells in a time-dependent manner. Compared to free Cou-6, the green fluorescence intensity of Cou-6-labeled PTX Emul was markedly enhanced at a predetermined time point, suggesting that PTX Emul was more readily internalized by 4T1 cells (Fig. 3B). The mean fluorescence intensity (MFI) of Cou-6-labeled PTX Emul-treated 4T1 cells was significantly increased compared to that of free Cou-6, consistent with the results observed with CLSM (Fig. 3C). To explore whether the ability of PTX Emul to enhance cellular uptake is mediated by LDL receptor, excessive LDL was added to cell medium to observe whether the cellular uptake of PTX Emul would be competitively inhibited. As shown in Fig. 3C, the MFI of Cou-6-labeled PTX Emul-treated 4T1 cells decreased significantly upon the addition of excess LDL. This may be due to the high expression of the LDL receptor on 4T1 cells, which facilitates the specific binding of PTX Emul to the LDL receptor and subsequent internalization into 4T1 cells. The addition of LDL competitively binds to the LDL receptor with PTX Emul, thereby inhibiting the LDL receptor-mediated cellular uptake profile of PTX Emul. CLSM images also confirmed these results, showing colocalization of PTX Emul (green) with endogenous LDL (red) on the cell membrane after incubation with 4T1 cells, further confirming the LDL receptor-mediated uptake pathway of PTX Emul into the cytoplasm (Fig. 3D). These results collectively point to PTX Emul being efficiently internalized by 4T1 cells via the LDL receptor-mediated active targeting mechanism, exhibiting excellent in vitro TNBC cell-targeted cellular uptake characteristics (Fig. 3E).

**Fig. 4.**
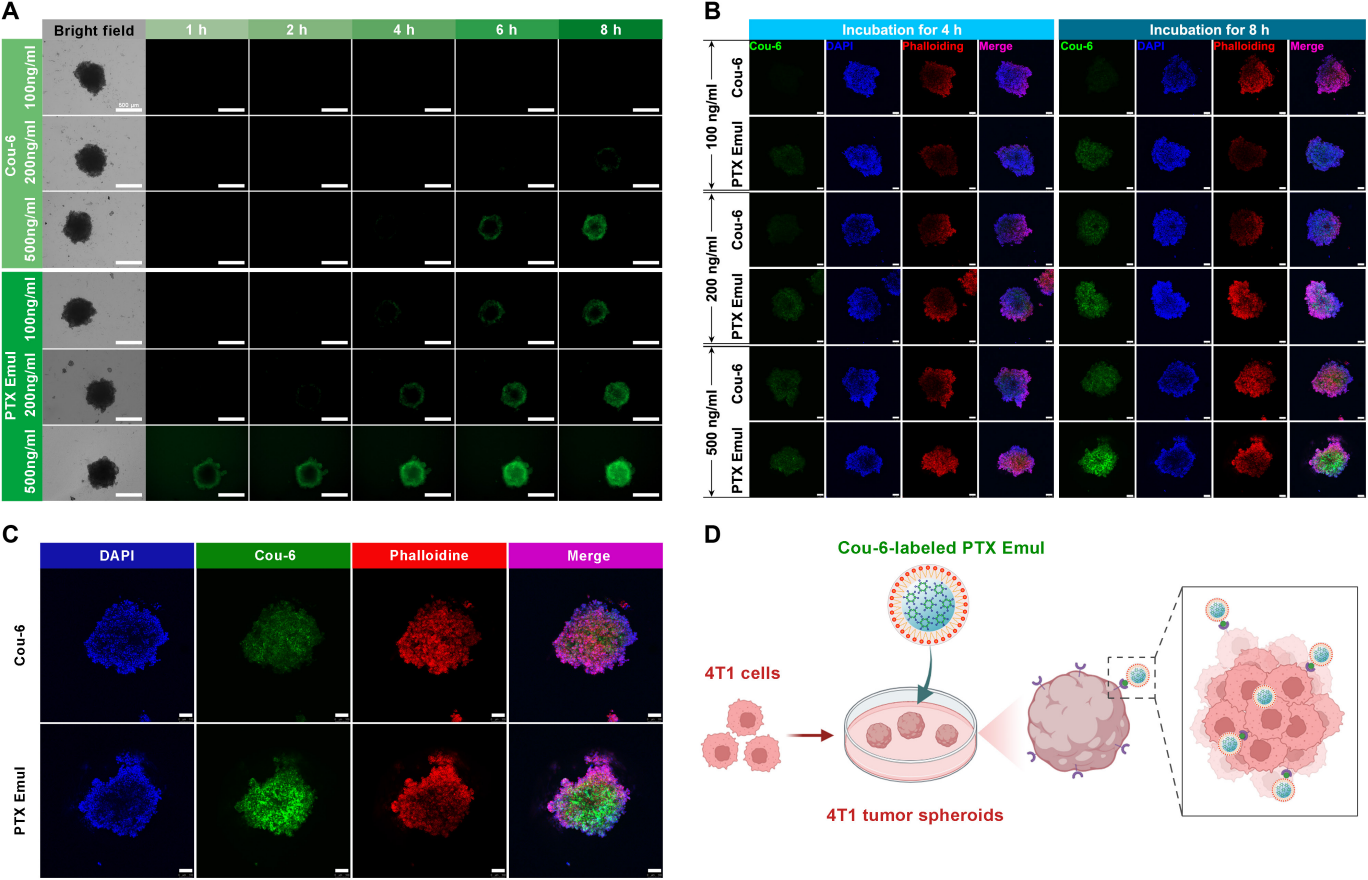
The penetration efficacy of PTX Emul labeled with Cou-6 in 4T1-derived 3D tumor spheroids. (A) Bright-field and fluorescence images of 4T1-derived 3D tumor spheroids after incubation with Cou-6 or Cou-6-labeled PTX Emul (green) at 100, 200, and 500 ng/ml for different durations (1, 2, 4, 6, and 8 h). Scale bar = 500 μm. (B) Representative CLSM photographs of 4T1-derived 3D tumor spheroids after incubation with Cou-6 or Cou-6-labeled PTX Emul (green) at 100, 200, and 500 ng/ml for 4 and 8 h. Scale bar = 100 μm. (C) Enlarged CLSM photographs of 4T1 cells after incubation with Cou-6 or Cou-6-labeled PTX Emul (green) at 500 ng/ml for 8 h. Scale bar = 100 μm. (D) Schematic illustrating the efficient penetration of PTX Emul into 4T1-derived 3D tumor spheroids via LDL receptor-mediated active targeting.

As compared with monolayer cells, 3-dimensional (3D) tumor spheroids grown from 4T1 cells possessed characteristics more representative of solid tumors in vivo. The targeted uptake behavior of PTX Emul was further investigated on the 4T1-derived 3D tumor spheroids model. As shown in Fig. 4A and B, a positive correlation was observed between Cou-6-labeled PTX Emul and both the time of incubation and concentration, indicating the effective accumulation of PTX Emul within 4T1-derived 3D tumor spheroids that increased over time and concentration. Under the same conditions in terms of incubation time and concentration, PTX Emul can markedly enhance the green fluorescence intensity in 3D tumor spheroids compared with free Cou-6, indicating that PTX Emul can penetrate 3D tumor spheroids more efficiently. Furthermore, PTX Emul efficiently penetrated the interior of tumor spheroids and exhibited uniform distribution (Fig. 4C). PTX Emul’s greater penetration efficiency in 4T1-derived 3D tumor spheroids is most likely attributable to its enhanced LDL receptor-mediated 4T1 targeted uptake profile (Fig. 4D). The efficient cellular uptake into 4T1 monolayer cells and 3D tumor spheroids lays a solid foundation for PTX Emul to exploit its ICD effect.

### The enhanced in vitro antitumor activity of PTX Emul

**Fig. 5.**
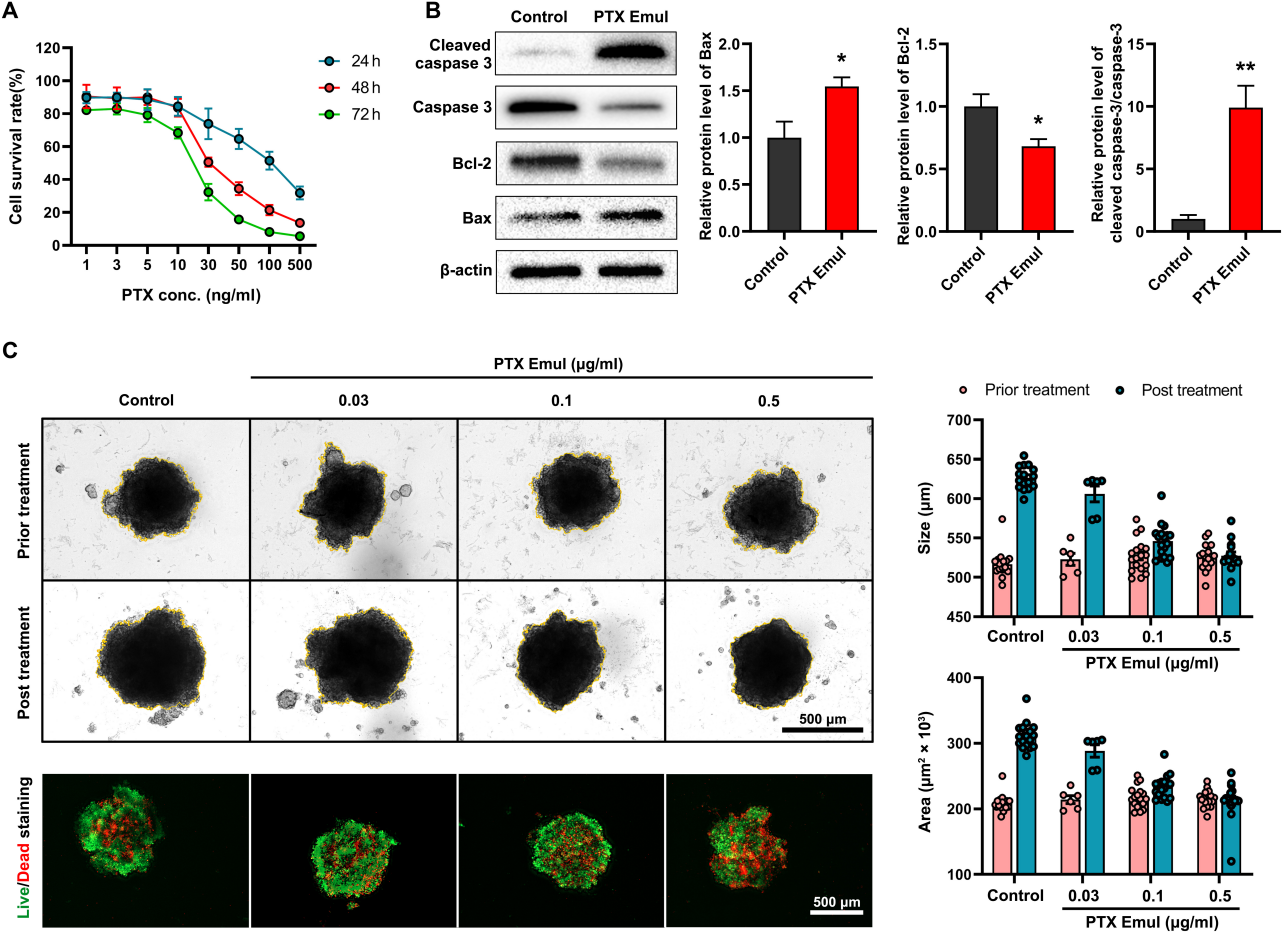
Effects of PTX Emul on the cell survival rate, apoptosis-related genes, and the growth of 4T1-derived tumor spheroids. (A) The survival percentage of 4T1 cells after incubation with PTX Emul for different durations. *n* = 4 to 6. (B) The relative expression levels of apoptosis-related proteins in 4T1 cells after 24 h treatment. *n* = 3. In comparison to the control, *P < 0.05 and **P < 0.01. (C) In vitro live/dead cell staining and growth measurement of 4T1-derived 3D tumor spheroids treated with 0.03, 0.1, or 0.5 μg/ml PTX Emul for 24 h. Scale bar = 500 μm. *n* = 6 to 15. All data are expressed as mean ± SEM.

To evaluate the antitumor activities of PTX Emul in vitro, assays were performed on cell viability, apoptosis-related genes, and the growth inhibition of 3D tumor spheroids. As the concentration of PTX Emul increased, the cell survival rate decreased, and this downward trend had a cumulative effect over time, with the lowest survival rate of 4T1 cells at 72 h (Fig. 5A). Simultaneously, the relative expression levels of representative apoptosis-related proteins were measured using Western blot after a 24-h treatment with PTX Emul at a concentration of 0.1 μg/ml. The treatment induced a dysregulation of the intrinsic apoptotic pathway, as indicated by a marked induction of the pro-apoptotic protein Bax and a significant suppression of the anti-apoptotic protein Bcl-2. Furthermore, the activation of the caspase cascades was confirmed by the increased level of cleaved caspase 3, accompanied by a decrease in its precursor caspase 3. These results collectively suggest that PTX Emul effectively induces apoptosis in 4T1 cells in vitro (Fig. 5B). Besides, we also quantitatively analyzed the morphological changes of 4T1-derived 3D tumor spheroids before and after treatment with 0.03, 0.1, or 0.5 μg/ml PTX Emul by measuring the diameter and area of tumor spheroids. As shown in Fig. 5C, PTX Emul markedly restricted the growth of 4T1-derived 3D tumor spheroids, as reflected by changes in their diameter and area, and the inhibitory effect exhibited a dose-dependent manner. The live/dead staining assay further confirmed that PTX Emul markedly induced apoptosis in tumor spheroids and exhibited a positive correlation with the concentration of PTX Emul. The enhancement in antitumor efficacy benefits from the improvements in cellular uptake of PTX Emul, as evidenced by the results from Fig. 3.

### The improved ICD induction and DCs maturation by PTX Emul in vitro

In the field of cancer immunotherapy, ICD has emerged as a key mechanism in eliciting antitumor immune response. ICD triggers the release of DAMPs, including the surface exposure of CRT, secretion of HMGB1, and extracellular release of ATP. These molecules act as “eat me” signals that are recognized by DCs, promoting DC maturation and enhancing their antigen-presenting capacity. This process subsequently activates a robust T cell-mediated antitumor immune response [15]. Accumulated evidence demonstrated that classical chemotherapeutic agents, such as PTX [36], can stimulate the body’s antitumor immune response, thereby inhibiting tumor growth through the induction of ICD. However, the use of free chemotherapeutic agents alone is not sufficient to induce an adequate ICD due to the lack of tumor-specific targeting capability, which substantially limits the effectiveness of ICD-based tumor immunotherapy [16]. In the current study, PTX Emul was developed as a TNBC-targeting delivery vehicle capable of precisely delivering PTX to TNBC cells via the LDL receptor-mediated active targeting pathway, as evidenced by the above results (Figs. 3 and 4). This facilitates the cellular accumulation of PTX in TNBC cells and promotes apoptosis more effectively, which is expected to improve the efficiency of ICD induction.

**Fig. 6.**
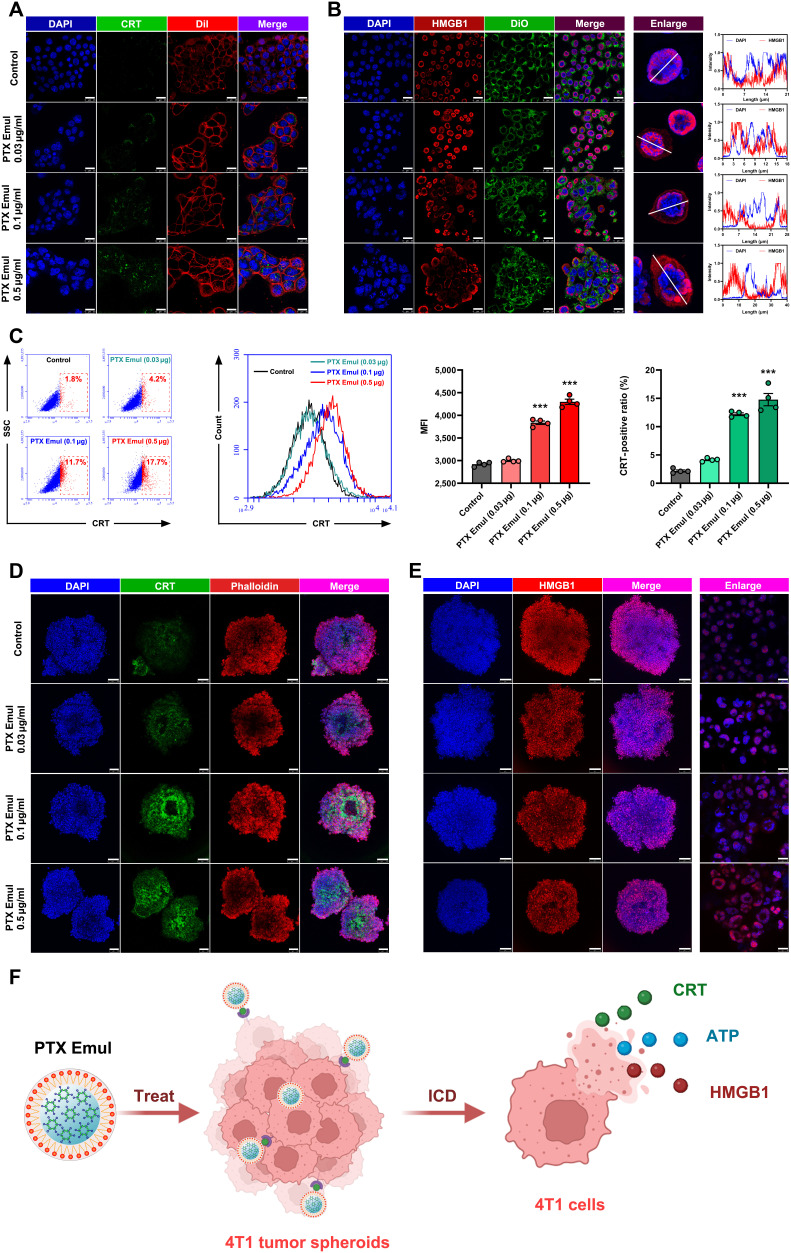
Enhanced ICD induction by PTX Emul in 4T1 monolayer cells and 4T1-derived tumor spheroids. (A) Representative CLSM images showing CRT exposure in 4T1 cells after 24-h treatment with PTX Emul. Scale bar = 25 μm. (B) Representative CLSM images and corresponding fluorescence intensity of HMGB1 release in 4T1 cells incubated with PTX Emul for 24 h. Scale bar = 25 μm. (C) Percentage of CRT-positive cells and mean fluorescence intensity in 4T1 cells following 24-h incubation with PTX Emul (*n* = 4). Data are presented as mean ± SEM. ***P < 0.001 versus control group. (D) Representative CLSM images of CRT exposure in 4T1-derived tumor spheroids treated with PTX Emul for 24 h. Scale bar = 100 μm. (E) Representative CLSM images of HMGB1 release in 4T1-derived tumor spheroids after 24-h treatment with PTX Emul. Scale bar = 75 μm. (F) Increased cellular uptake of PTX Emul via the LDL receptor-mediated active targeting pathway potentiated ICD induction, as demonstrated by elevated CRT exposure and enhanced secretion of HMGB1 and ATP.

The exposure of CRT, the secretion of HMGB1, and the release of ATP from dying tumor cells are established as hallmarks of ICD [16]. In the concentration range of PTX Emul from 0.03 to 0.5 μg/ml, PTX Emul is capable of markedly enhancing CRT exposure on the 4T1 cell surface and promoting the transfer of HMGB1 from the nucleus to the cytoplasm, with an obvious dose–effect relationship (Fig. 6A and B). The quantitative results of CRT exposure on the surface of 4T1 cells after administration were further analyzed by flow cytometry. Compared with the control group, CRT exposure in the PTX Emul (0.1 μg/ml) and PTX Emul (0.5 μg/ml) groups was significantly enhanced, as evidenced by the increased MFI and CRT positive ratio (Fig. 6C). Additionally, the ATP and HMGB1 levels released by 4T1 cells were assessed quantitatively by ELISA kits after treatment with PTX Emul. As shown in Figs. S1 and S2, both 0.1 and 0.5 μg/ml PTX Emul-treated 4T1 cells generated higher levels of ATP and HMGB1 as compared to those of the control. Comparable findings were observed through Western blot analysis. Specifically, PTX Emul (0.1 μg/ml) and PTX Emul (0.5 μg/ml) groups showed a significant increase in CRT protein expression levels relative to the control, while HMGB1 protein expression levels were lower (Figs. S3 and S4). In addition to 4T1 monolayer cells, the ICD-inducing capability of PTX Emul on 4T1-derived 3D tumor spheroids was explored. Similar to the results obtained with 4T1 monolayer cells, PTX Emul effectively induces characteristic signatures of ICD, including CRT exposure and release of HMGB1 in 3D tumor spheroids (Fig. 6D and E). In summary, the elevated levels of CRT exposure and secretion of HMGB1 and ATP from the PTX Emul-treated 4T1 monolayer cells or 3D tumor spheroids confirmed the more efficient ICD, which might be linked to better cellular uptake of PTX Emul (Fig. 6F).

**Fig. 7.**
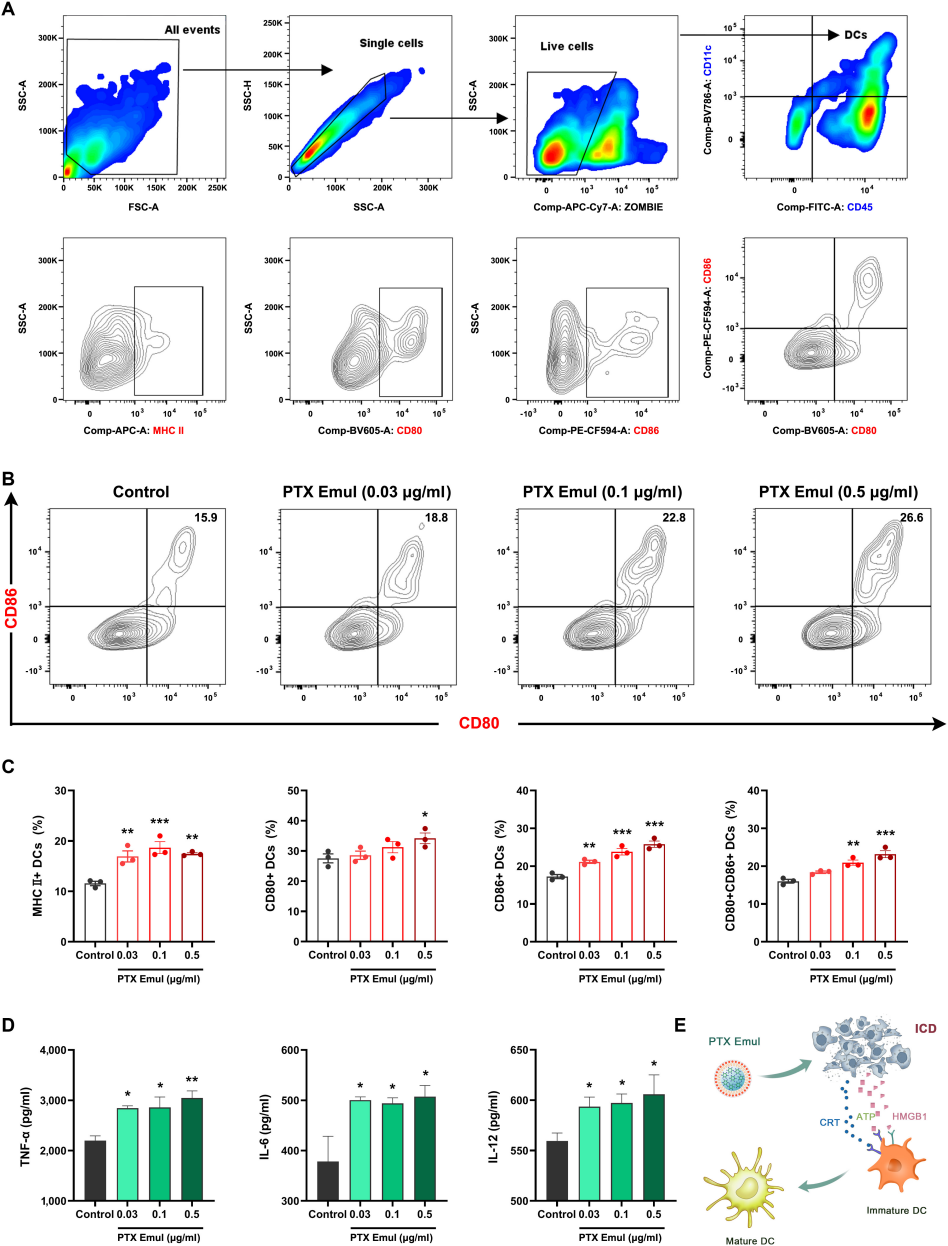
The enhanced BMDCs maturation by PTX Emul in vitro. (A and B) Representative flow cytometry plots of BMDC maturation elicited by PTX Emul. (C) Flow cytometry analysis of MHC II^+^, CD80^+^, CD86^+^, and CD80^+^CD86^+^ DCs (in the CD11c^+^ gate) in different groups. Data are presented as the mean ± SEM (*n* = 3). *P < 0.05, **P < 0.01, and ***P < 0.001 compared with the control group. (D) Pro-inflammatory cytokines, including TNF-α, IL-6, and IL-12, are secreted by BMDCs after treatment with PTX Emul. Data are presented as the mean ± SEM (*n* = 3). *P < 0.05 and **P < 0.01 compared with the control group. (E) PTX Emul promoted DCs activation by inducing the ICD effect in 4T1 cells.

Upon ICD induction, tumor antigens released through this process can serve as “eat me” signals that promote the maturation of DCs. Accordingly, the maturation of DCs triggered by PTX Emul-treated 4T1 cells was assessed via flow cytometry (Fig. 7A and B). As depicted in Fig. 7C, all tested concentrations of PTX Emul significantly enhanced the surface expression of MHC II and CD86 on BMDCs. Notably, PTX Emul at 0.5 μg/ml further increased the expression of MHC II, CD80, and CD86, leading to the most pronounced effect in stimulating the maturation of BMDCs, as indicated by the elevated population of CD80^+^CD86^+^ mature DCs. Therefore, PTX Emul could induce robust ICD effects for the high potential of cancer immunotherapy. In line with these findings, PTX Emul at all tested concentrations significantly stimulated BMDCs to secrete key cytokines—tumor necrosis factor-α (TNF-α), interleukin-6 (IL-6), and IL-12 (Fig. 7D). The findings indicate that PTX Emul effectively induces ICD in 4T1 cells, leading to the release of DAMPs, such as CRT exposure, HMGB1 secretion, and ATP release. These DAMPs function as “eat me” signals to DCs, subsequently promoting their maturation (Fig. 7E).

### Tumor-targeting biodistribution of PTX Emul and MLN-targeting biodistribution of CHA-SME in vivo

**Fig. 8.**
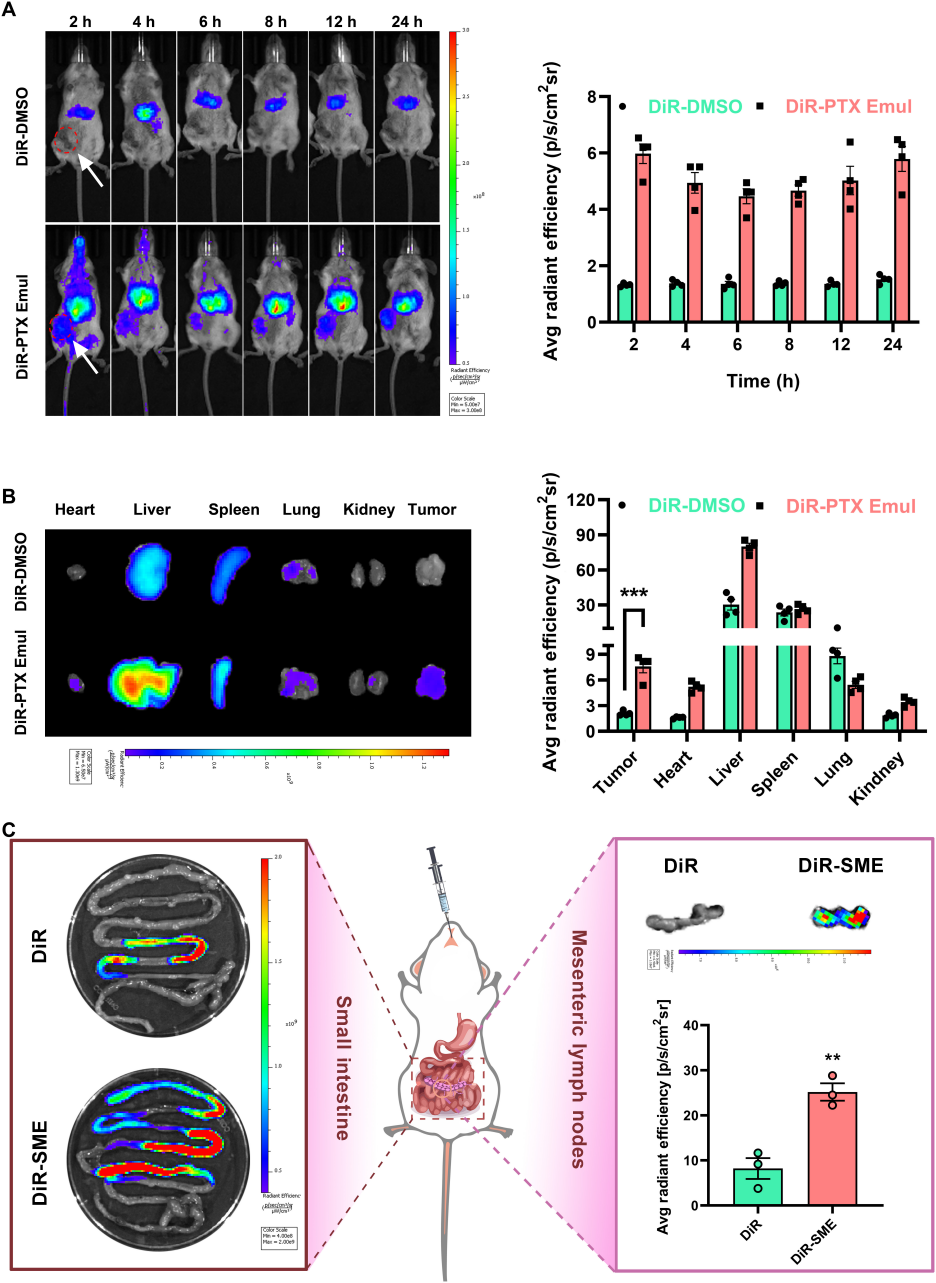
In vivo biodistribution of DiR-labeled PTX Emul (intravenous) and DiR-labeled CHA-SME (oral) in orthotopic 4T1 tumor-bearing BALB/c mice. (A) In vivo fluorescence imaging of the distribution of DiR-DMSO and DiR-labeled PTX Emul in mice at predetermined time points (2, 4, 6, 8, 12, and 24 h) after intravenous administration and semiquantitative analysis of the tumor region. Data are presented as the mean ± SEM (*n* = 4). (B) Ex vivo fluorescence images and semiquantitative analysis of DiR-DMSO and DiR-labeled PTX Emul in tumors and organs at 24 h after intravenous administration. Data are presented as the mean ± SEM (*n* = 4). ***P < 0.001. (C) Ex vivo fluorescence images of DiR-DMSO and DiR-labeled CHA-SME in the intestine and MLNs at 2 h after oral administration, and semiquantification. Data are presented as the mean ± SEM (*n* = 4). **P < 0.01.

The in vivo tumor-targeting biodistribution of PTX Emul and MLN-targeting biodistribution of CHA-SME were determined by the near-infrared fluorescence imaging in orthotopic 4T1 tumor-bearing BALB/c mice. To assess the tissue distribution characteristics of PTX Emul and CHA-SME, DiR was used as the near-infrared fluorescent dye, and DiR-labeled PTX Emul and CHA-SME were prepared for in vivo tracing. As shown in Fig. 8A, free DiR was mainly distributed in the liver at 2 h after injection and did not display apparent distribution in the tumor until 24 h. In contrast, obvious fluorescence was observed in the tumor tissue within 2 h after injection of DiR-labeled PTX Emul, and the fluorescence accumulation remained stable for 24 h, indicating that PTX Emul can be rapidly and stably targeted to the tumor tissue after entering the blood circulation. Notably, tumors in the DiR-labeled PTX Emul group exhibited approximately 3.3 times higher fluorescence intensity than those in the DiR-DMSO control group at all time points examined. Ex vivo fluorescence images from the dissected tumors revealed similar results, with tumors from the DiR-labeled PTX Emul group exhibiting significantly greater fluorescence intensity than tumors from the DiR-DMSO group (Fig. 8B). The findings suggest that PTX Emul exhibits effective tumor targeting in orthotopic 4T1 tumor-bearing mice, potentially due to the EPR effect in conjunction with the active targeting pathway mediated by LDL receptors.

SMEs have garnered increasing interest for their ability to target MLNs via the intestinal lymphatic transport pathway [26]. As illustrated in Fig. 8C, ex vivo fluorescence imaging demonstrated markedly higher fluorescence intensity in the small intestine of mice treated with DiR-labeled CHA-SME compared to those receiving DiR-DMSO, at 2 h after oral administration. Notably, the fluorescence signal in the MLNs of the CHA-SME group was 2.8-fold stronger than that in the DiR-DMSO group. These results suggest that CHA-SME not only enhances the gastrointestinal absorption of its cargo but also effectively directs it to the MLNs, facilitating drug accumulation within these lymphoid tissues via the lymphatic route. Collectively, the findings support that CHA-SME serves as an efficient targeted delivery platform for transporting immunomodulators to MLNs.

Owing to their distinct targeting profiles—PTX Emul for tumors and CHA-SME for MLNs—in orthotopic 4T1 tumor-bearing mice, PTX and CHA can be delivered precisely to the tumor microenvironment and MLNs, respectively. Thereby, this strategy not only induces ICD efficiently at the tumor site but also promotes immune cell activation in the MLNs, leading to a potentiated antitumor immune response.

### The superior in vivo antitumor efficacy of PTX Emul combined with CHA-SME

**Fig. 9.**
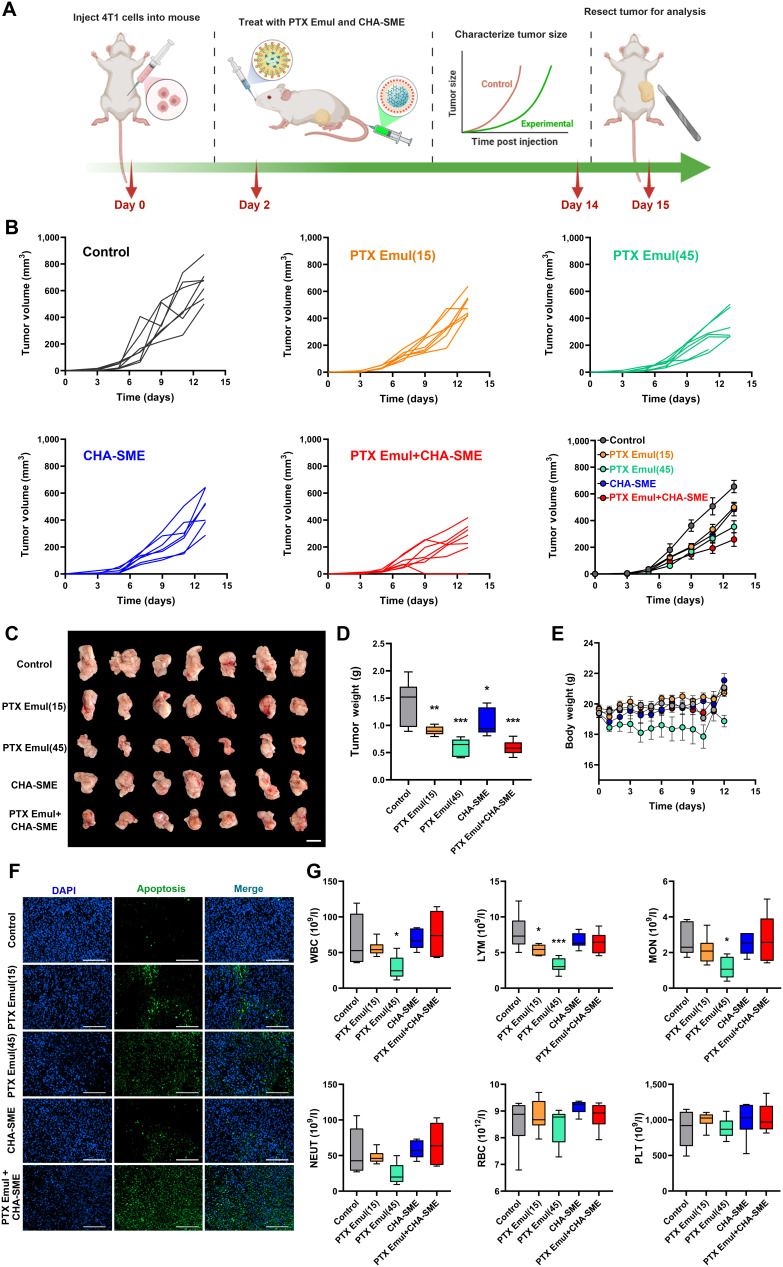
In vivo antitumor efficacy of the dual-pronged approach of TNBC-targeted PTX Emul and lymph node-targeted CHA-SME in an orthotopic 4T1 murine TNBC model. (A) Schematic diagram of the treatment process. (B) Tumor growth curves of different treatment groups, including PTX Emul (15 mg/kg), PTX Emul (45 mg/kg), CHA-SME (35 mg/kg), and PTX Emul (15 mg/kg) plus CHA-SME (35 mg/kg). *n* = 7 per group. (C) Representative images of excised tumors. Scale bar = 1 cm. (D) Weights of excised tumors. *n* = 7 per group. *P < 0.05, **P < 0.01, and ***P < 0.001 versus control. (E) Body weight changes of mice during treatment. *n* = 7 per group. (F) TUNEL staining of tumor tissues. Scale bar = 100 μm. (G) Hematological parameters, including WBC, LYM, MON, NEUT, RBC, and PLT levels. *n* = 6 per group. Data are mean ± SEM. *P < 0.05 and ***P < 0.001 versus control.

Prompted by the enhanced ICD induction and DC maturation by PTX Emul, along with the demonstrated targeting efficiency of PTX Emul and CHA-SME both in vitro and in vivo, we further evaluated the synergistic antitumor efficacy of PTX Emul combined with CHA-SME in orthotopic 4T1 tumor-bearing mice (Fig. 9A). To assess the therapeutic outcomes of chemotherapy and immunotherapy, the following 5 experimental groups were established: PBS control, high-dose PTX Emul (45 mg/kg), low-dose PTX Emul (15 mg/kg), CHA-SME (35 mg/kg), and low-dose PTX Emul (15 mg/kg) plus CHA-SME (35 mg/kg). Numerous studies have demonstrated that chemotherapy generally exhibits immunosuppressive effects, primarily due to its cytotoxic impact on proliferating cells within the bone marrow and peripheral lymphoid tissues, particularly when administered at the maximum tolerated dose (MTD) [15,22,30]. Lymphopenia is regarded as the most pronounced immunosuppressive adverse effect. In our prior investigation, we determined that the MTD of PTX Emul in BALB/c nude mice was 45 mg/kg. At this dose, PTX Emul reduced T cell subpopulations and impaired DC maturation in both the peripheral blood and spleen [22]. Therefore, leveraging the tumor-targeting advantage of PTX Emul, the use of a low dose (15 mg/kg) effectively induces ICD within the tumor microenvironment. This approach not only circumvents the immunosuppressive effects associated with high-dose chemotherapy (45 mg/kg) but also activates an antitumor immune response through the ICD mechanism. Furthermore, our previous study showed that orally administered CHA-SME (35 mg/kg) significantly suppresses tumor growth. This antitumor effect is mediated by promoting DC maturation and priming naïve T cells toward effector T cell differentiation [26]. Based on these findings, doses of 15 mg/kg for PTX Emul and 35 mg/kg for CHA-SME were selected for evaluating the in vivo antitumor efficacy of the combined chemotherapy and immunotherapy treatment.

As shown in Fig. 9B to D, all treated groups in the orthotopic 4T1 model exhibited a significant reduction in both tumor volume and tumor weight compared with the control group, confirming their antitumor efficacy. Compared to the low-dose (15 mg/kg) PTX Emul, PTX Emul at the MTD (45 mg/kg) showed superior antitumor efficacy and obvious weight reduction (Fig. 9E), which was attributed to the dose-enhancing effect of the cytotoxic agent. Similar to the low-dose (15 mg/kg) PTX Emul, CHA-SME alone only slightly inhibited 4T1 tumor growth. These results showed that neither low-dose PTX Emul nor CHA-SME alone could achieve the desired antitumor efficacy, indicating that the ICD effect within the tumor microenvironment or the immune cell activation within the MLNs alone is not sufficient to counteract rapid tumor growth. Interestingly, low-dose PTX Emul (15 mg/kg) significantly inhibited tumor growth in combination with CHA-SME, comparable to PTX Emul at the MTD (45 mg/kg). For further evaluation of the therapeutic effects, tumors were harvested for observation of tumor cell apoptosis in different groups by terminal deoxynucleotidyl transferase-mediated deoxyuridine triphosphate nick end labeling (TUNEL) assay. As shown in Fig. 9F, the groups treated with PTX Emul (45 mg/kg) and PTX Emul (15 mg/kg) plus CHA-SME showed markedly more intense apoptotic fluorescence signals (green) among all treatment groups, which was consistent with the decreased tumor volume and tumor weight.

One of the most important prerequisites for a potential combination of chemotherapy and immunotherapy is that chemotherapy does not impair the body’s immune function [15]. To assess the potential immunosuppressive effects of PTX Emul, peripheral blood samples were subjected to complete blood cell count analysis. As shown in Fig. 9G, treatment with CHA-SME or the combination of PTX Emul and CHA-SME did not alter the levels of WBC, LYM, MON, NEUT, RBC, and PLT. In addition to the slight decrease in LYM, low-dose (15 mg/kg) PTX Emul did not affect the other hematologic markers observed. Nevertheless, administration of PTX Emul at the MTD (45 mg/kg) resulted in a marked reduction in the levels of WBC, LYM, MON, and NEUT in peripheral blood. This suggests that PTX Emul at the MTD can substantially decrease circulating lymphocyte levels. Lymphocytes, typically represented by T cells, B cells, and DCs, are a dominant immune population involved in antitumor immune responses [37]. The reduction in circulating lymphocytes from the peripheral blood may impair the antitumor activity of the immune system [38]. These results suggested that low-dose (15 mg/kg) PTX Emul has a minimal immunosuppressive effect compared to PTX Emul (45 mg/kg). Our findings regarding the dose-dependent effects of PTX Emul align with the well-established “double-edged sword” paradigm of chemotherapy-induced immunomodulation. While high-dose, maximum-tolerated chemotherapy often causes systemic immunosuppression—primarily through lymphodepletion and impaired DC function—emerging evidence indicates that lower, metronomic doses can more effectively induce ICD and stimulate antitumor immunity without compromising the host immune system [15,22,39–42]. This concept is supported by studies demonstrating that optimized low-dose regimens of agents such as oxaliplatin promote ICD and immune activation, whereas high doses abrogate these effects [43]. In our study, the low dose of PTX Emul (15 mg/kg) robustly induced ICD hallmarks without pronounced lymphopenia, whereas the high dose (45 mg/kg) showed attenuated ICD signals alongside reduced lymphocyte counts, corroborating this dose-dependent duality. Therefore, the selection of a low, immunogenic dose of PTX Emul, combined with lymphoid-targeted immunomodulation via CHA-SME, represents a rational strategy to harness the immunostimulatory potential of chemotherapy while mitigating its immunosuppressive edge. Based on these findings, the enhanced antitumor efficacy of the PTX Emul and CHA-SME combination can be attributed to PTX Emul-induced ICD and the subsequent amplification of ICD-associated antitumor immunity by CHA-SME.

Additionally, the in vivo toxicity of PTX Emul plus CHA-SME was investigated after the treatment. The concentrations of blood urea nitrogen, creatinine, uric acid, and aspartate aminotransferase were within normal physiological ranges, suggesting an absence of damage to the liver, kidneys, and heart, respectively (Fig. S5). Furthermore, histopathological examination of major organs revealed no notable abnormalities or damage post-treatment (Fig. S6). These findings demonstrated that the dual-pronged approach of TNBC-targeted PTX Emul and lymph node-targeted CHA-SME was safe for in vivo immunochemotherapy of TNBC.

It is important to note that while the immunocompetent 4T1 syngeneic mouse model provides a valuable system for studying tumor-immune interactions and initial proof of concept, it does not fully recapitulate the genomic complexity, stromal heterogeneity, and specific immune cell repertoire of human TNBC. These inherent differences limit the direct extrapolation of our findings to the clinical setting. Therefore, future validation in more clinically relevant models is essential. Subsequent studies should employ patient-derived xenograft (PDX) models, which preserve the histopathological and molecular characteristics of human tumors, and/or humanized mouse models reconstituted with a human immune system. These advanced preclinical models will be critical for assessing whether the potent antitumor and immunomodulatory effects observed with our PTX Emul + CHA-SME combination translate to a human context, thereby strengthening the rationale for its potential clinical development.

### The improved ICD induction by PTX Emul in vivo

**Fig. 10.**
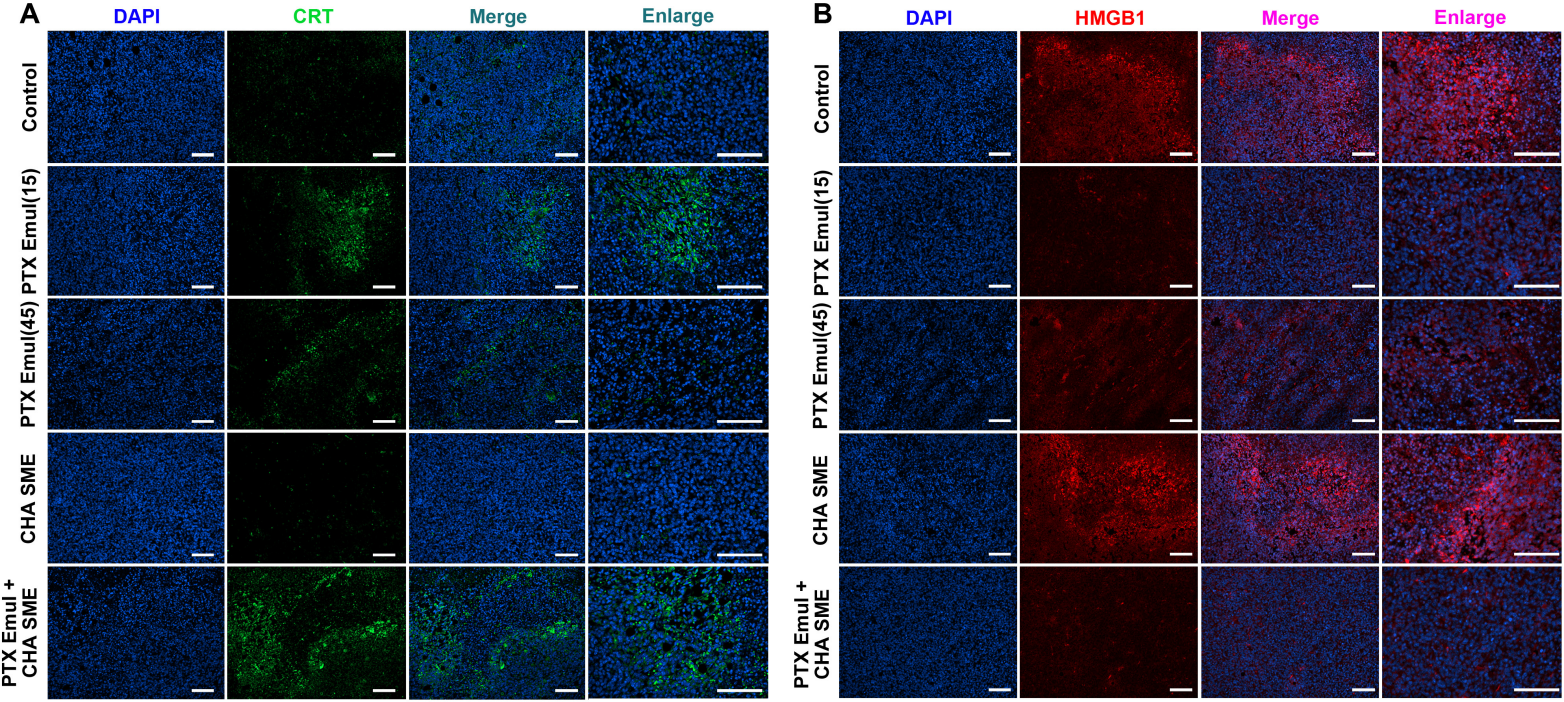
The improved ICD induction by the 2-pronged approach of PTX Emul plus CHA-SME in vivo. (A) Representative CLSM images showing CRT exposure in tumor tissues following treatment with PTX Emul (15 mg/kg), PTX Emul (45 mg/kg), CHA SME (35 mg/kg), or PTX Emul (15 mg/kg) plus CHA SME (35 mg/kg). CRT is shown in green, and nuclei were stained with DAPI (blue). Scale bar = 100 μm. (B) Representative CLSM images of HMGB1 release in tumor tissues under the same treatment conditions. HMGB1 is shown in red, and nuclei were stained with DAPI (blue). Scale bar = 100 μm.

To determine whether the enhanced antitumor efficacy of the combination therapy stems from the activation of ICD-associated antitumor immunity, we assessed ICD induction by PTX Emul by measuring the surface exposure of CRT and the extracellular release of HMGB1. As shown in Fig. 10A, both low-dose PTX Emul (15 mg/kg) and PTX Emul combined with CHA-SME markedly increased pre-apoptotic CRT exposure (green fluorescence) on the cell surface compared with the control and CHA-SME-alone groups. A similar trend was observed for HMGB1 translocation from the nucleus to the cytoplasm: tumor cells treated with low-dose PTX Emul (15 mg/kg) or the combination showed markedly reduced nuclear HMGB1 levels compared with those treated with PBS or CHA-SME alone (Fig. 10B). Notably, tumors treated with high-dose PTX Emul (45 mg/kg) exhibited less CRT exposure and HMGB1 translocation than those receiving low-dose PTX Emul (15 mg/kg). This is consistent with the established dose dependency of ICD induction by cytotoxic agents, where higher chemotherapy doses can indiscriminately kill tumor, normal, and immune cells, leading to systemic immunosuppression. It has been shown that relatively low doses of chemotherapeutic agents are usually more effective in inducing ICD and stimulating systemic antitumor immunity than high concentrations of chemotherapeutic agents [43]. Together, the observation of these 2 primary indicators of ICD demonstrated that either low-dose PTX Emul (15 mg/kg) or PTX Emul plus CHA-SME was highly efficient in triggering ICD activity within the tumor microenvironment of 4T1 orthotopic tumor, which created a prerequisite for activating subsequent ICD-associated antitumor immune responses.

### Enhancement of antitumor immune responses by PTX Emul combined with CHA-SME

**Fig. 11.**
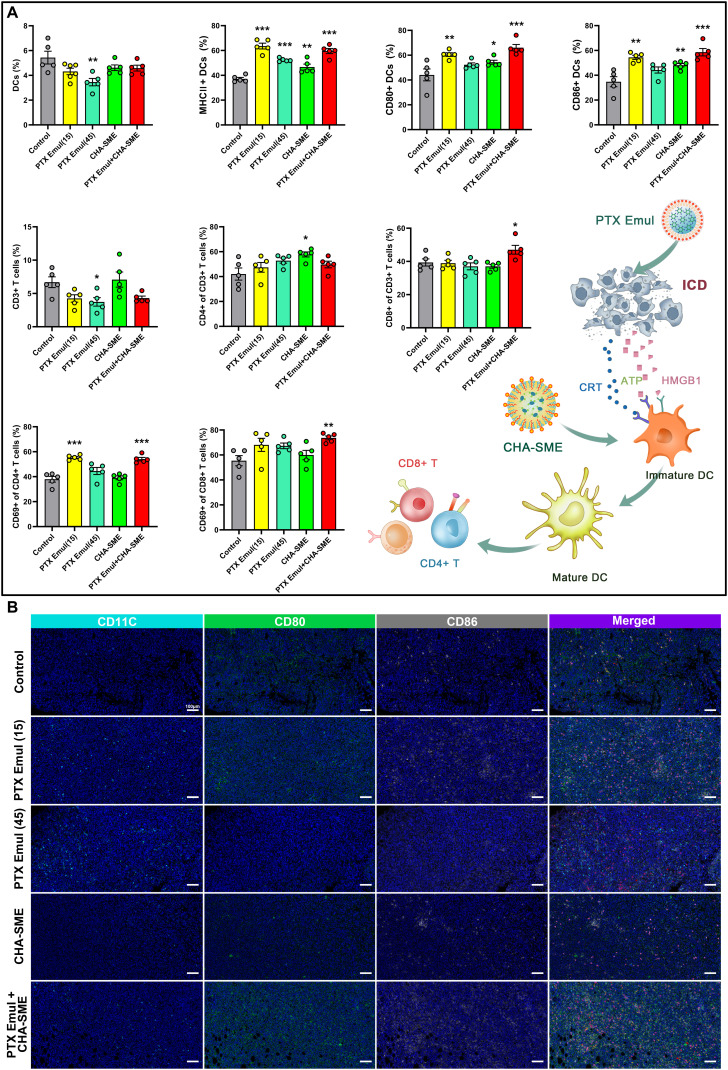
Enhancement of antitumor immune responses by the dual-pronged approach of TNBC-targeted PTX Emul and lymph node-targeted CHA-SME. (A) The proportions of CD11c^+^, CD11c^+^MHC II^+^, CD11c^+^CD80^+^, and CD11c^+^CD86^+^ mDCs infiltrated into the tumor. The proportions of CD3^+^, CD3^+^CD4^+^, CD3^+^CD8^+^, CD4^+^CD69^+^, and CD8^+^CD69^+^ T cells infiltrated into the tumors. Each value represents the mean ± SEM (*n* = 5). *P < 0.05, **P < 0.01, and ***P < 0.001 compared with the control group. (B) Multiplex immunofluorescent staining of DC maturation infiltrated in the tumors. Scale bar = 100 μm.

Inspired by the excellent antitumor efficacy and the improved ICD induction, we are keen to explore the extent to which combination therapy strategies activate antitumor immunity in vivo. Given that PTX Emul-induced ICD leads to the release of DAMPs, which subsequently promote DC maturation, we analyzed the surface expression of mature DC markers (MHC II, CD80, and CD86) in isolated tumor tissues by flow cytometry. By calculating the proportion of DCs in the tumor microenvironment, only PTX Emul at the MTD (45 mg/kg) was found to attenuate the proportion of DCs, further confirming that the MTD of chemotherapeutic drugs kills immune cells and weakens the body’s immune system. As expected, treatment with low-dose PTX Emul (15 mg/kg), CHA-SME, and PTX Emul plus CHA-SME elevated the expression of all surface markers of DC maturation, including MHC II, CD80, and CD86. Among these, PTX Emul plus CHA-SME has the greatest ability to promote DC maturation, which may be attributed to the improved ICD induction by low-dose PTX Emul and the activation of immune cells after targeting MLNs by CHA-SME. In contrast, the ability of high-dose PTX Emul (45 mg/kg) to promote DC maturation was weaker, with only an increase in MHC II expression (Fig. 11A), which is associated with immunosuppression from high-dose chemotherapy. Moreover, analysis of multiple immunofluorescence staining also confirmed that the PTX Emul plus CHA-SME-treated group had the highest percentage of CD80 and CD86 positivity within the tumor microenvironment compared to the other treatment groups (Fig. 11B).

The above findings demonstrated that the activation of ICD by PTX-Emul markedly promoted DC maturation. However, whether mature DCs can effectively present tumor antigens to activate T cells and thereby generate robust T cell-mediated antitumor immunity remains to be fully elucidated. Consequently, we analyzed the tumor-infiltrating T cell population within the tumors to further examine the extent of immune activation induced by PTX Emul in combination with CHA-SME. While high-dose PTX Emul (45 mg/kg) substantially reduced the proportion of CD3^+^CD4^+^ T cells, neither low-dose PTX Emul nor PTX Emul plus CHA-SME induced pronounced changes (Fig. 11A). CHA-SME monotherapy significantly increased the proportion of CD3^+^CD4^+^ T cells, likely due to T cell activation following targeted delivery of CHA to the MLNs—a finding consistent with previous reports [26]. In contrast, the combination of PTX Emul and CHA-SME markedly enhanced the infiltration of cytotoxic CD3^+^CD8^+^ T cells in tumors compared with the control group. This result was further supported by multiplex immunofluorescence staining of tumor tissues, which showed the strongest CD3^+^CD8^+^ signal in the PTX Emul plus CHA-SME group (Fig. S7). Moreover, elevated levels of activated T cells, reflected by CD4^+^CD69^+^ and CD8^+^CD69^+^ populations, were detected in tumors of the combination-treated mice, indicating effective induction of T cell-mediated antitumor immunity. CD69 is an early activation marker expressed on various leukocyte subsets, including T cells and natural killer (NK) cells, and plays a key role in regulating antitumor immune responses [44,45]. When assessing CD69 expression on the surface of CD8^+^ or CD4^+^ T cells to monitor early T cell activation, the CD4^+^CD69^+^ population indicates early activation of CD4^+^ T cells, whereas the CD8^+^CD69^+^ population indicates early activation of CD8^+^ T cells [46,47]. Thus, the increase in CD69 expression on CD4^+^ and CD8^+^ T cells is critical for triggering potent antitumor immune responses. Further cytokine analysis supported these findings. Treatment with PTX Emul plus CHA-SME up-regulated the secretion of IL-1α, TNF-α, and interferon-β (IFN-β) (Fig. S8)—pro-inflammatory cytokines known to promote the recruitment and activation of both innate and adaptive immune cells, such as macrophages, DCs, and cytotoxic T lymphocytes. In summary, the markedly enhanced antitumor immunity observed with the combination therapy stems from a synergistic interplay: PTX Emul induces ICD within the tumor, while CHA-SME potentiates immune activation in the MLNs.

While this study primarily delineates the DC–T cell axis as the central effector mechanism activated by our dual-targeting strategy, we acknowledge the integral roles played by other immune populations within the complex tumor microenvironment. For instance, NK cells are potent mediators of innate antitumor cytotoxicity and can be activated by cytokines (e.g., IL-12 and IFN-γ) secreted by mature DCs and T helper 1 cells. Similarly, tumor-associated macrophages (TAMs), particularly the repolarization from a pro-tumorigenic M2 phenotype to an immunostimulatory M1 state, can markedly influence treatment efficacy. Furthermore, regulatory T cells (Tregs) are key mediators of immune suppression, and their modulation is critical for sustaining effective antitumor immunity. Our strategic focus on the DC–T cell axis was based on the established mechanistic paradigm wherein ICD, the cornerstone of our approach, primarily operates through the release of DAMPs that are sensed by DCs, leading to their maturation and subsequent priming of tumor-specific T cells. This pathway represents the most direct and well-characterized adaptive immune cascade initiated by ICD-inducing agents. The pronounced enhancement in DC maturation, CD8^+^ T cell infiltration, and effector function observed herein provides robust validation of this core mechanism. Future studies will employ comprehensive immune profiling to systematically quantify the dynamics of NK cells, macrophages, Tregs, and other myeloid subsets in response to our combination therapy, thereby providing a more holistic view of the remodeled immune landscape.

Our “tumor + lymph node dual-targeting” strategy offers a mechanistically distinct approach compared to conventional chemo-immunotherapy regimens. Unlike traditional “chemotherapy + immune checkpoint inhibitor (ICI)” combinations—which often show limited efficacy in TNBC due to an immunosuppressive microenvironment and inadequate pre-existing T cell responses—our approach actively remodels the immune landscape. The tumor-targeted PTX Emul induces potent ICD, converting the tumor into an in situ vaccine, while the lymph node-targeted CHA-SME activates DCs and primes naïve T cells, thereby generating a de novo, systemic antitumor immune response. This coordinated intervention addresses 2 key barriers to ICI efficacy: insufficient antigen release and peripheral immune tolerance. Consequently, PTX Emul + CHA-SME can serve as an effective immune primer to sensitize cold tumors to ICIs, offering a rational combinatorial strategy to improve response rates. Furthermore, compared to “chemotherapy + systemic immune agonist” approaches that may cause off-target toxicity, our spatially targeted nanocarriers enable precise immune modulation at the critical anatomical sites governing antitumor immunity, potentially enhancing efficacy while minimizing systemic adverse effects.

### PTX Emul plus CHA-SME for inhibiting lung metastasis by stimulated systemic immune responses

**Fig. 12.**
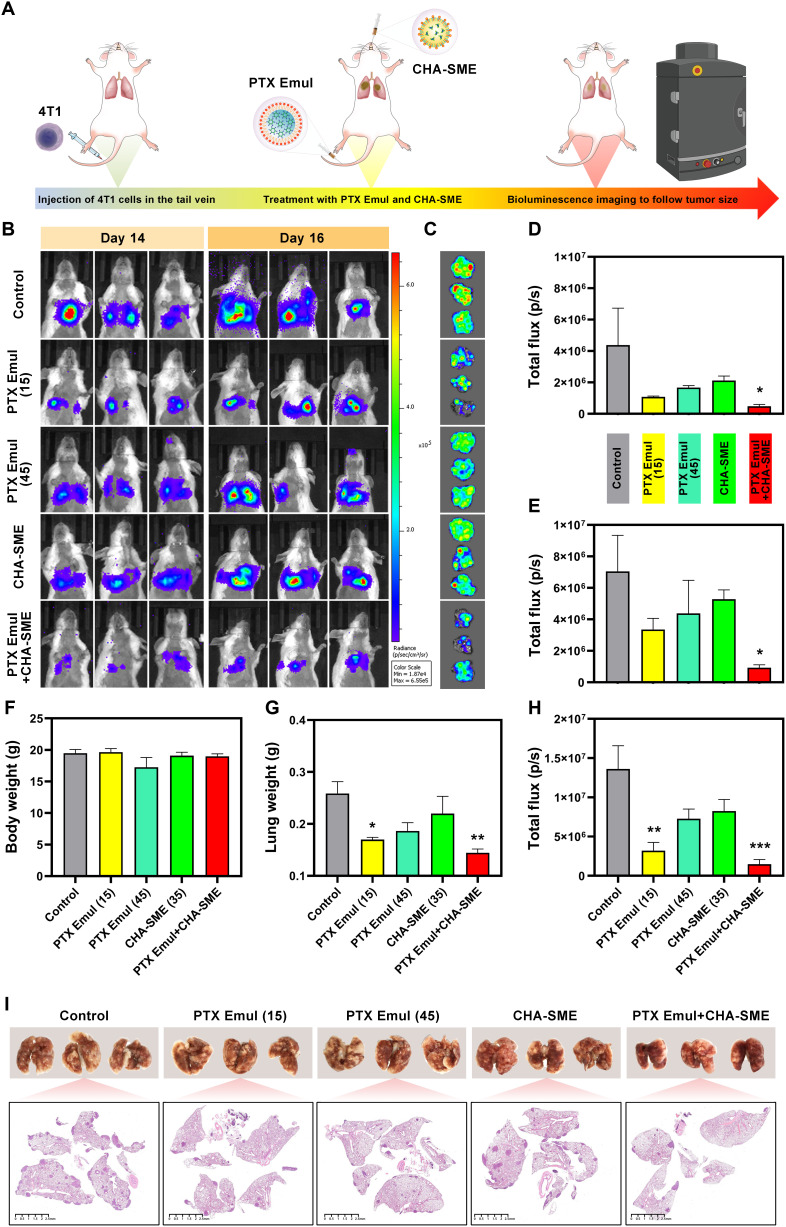
The dual-pronged approach of TNBC-targeted PTX Emul and lymph node-targeted CHA-SME for eradicating lung metastasis. (A) Schematic of the experimental design for evaluating anti-metastatic efficacy. (B) In vivo bioluminescence images of mice from each group on days 14 and 16. (C) Ex vivo bioluminescence images of lung tissues collected on day 16. (D) Total flux of the region of interest (ROI) measured on day 14. (E) Total flux of the ROI measured on day 16. (F) Body weights of mice across treatment groups recorded on day 16. (G) Lung weights of mice from each group on day 16. (H) Total flux of the excised lung tissues. (I) Photographs of lung tissues showing metastatic nodules and corresponding H&E-stained whole-lung sections. Data are presented as mean ± SEM (*n* = 3). *P < 0.05, **P < 0.01, AND ***P < 0.001 versus control.

Encouraged by the outstanding tumor inhibition and immune microenvironment regulation effect of PTX Emul plus CHA-SME, we explored the anti-metastasis capability of PTX Emul plus CHA-SME in the lung metastatic TNBC model. TNBC is characterized by high aggressiveness and a strong propensity for metastasis. Lung metastasis is particularly common in TNBC, occurring in 36.9% of patients with recurrent disease and contributing to a low 5-year survival rate. Metastasis is responsible for over 90% of TNBC-related mortality in women [48,49]. A lung metastatic TNBC model was established by intravenous injection of 4T1-Luc cells (Fig. 12A). The development and progression of lung metastases were monitored and compared using small-animal in vivo fluorescence imaging to assess fluorescence intensity in the lungs. Bioluminescence imaging revealed severe tumor metastasis in the control group, whereas only minimal metastatic signals were detected in the PTX Emul plus CHA-SME group on day 10 and day 14 (Fig. 12B). Although the PTX Emul (15 mg/kg), CHA-SME, and PTX Emul (45 mg/kg) could also reduce lung fluorescence intensity to some extent, their ability to inhibit lung metastasis was markedly weaker than that of the PTX Emul plus CHA-SME group (Fig. 12D and E). Bioluminescence imaging was followed by lung isolation, weighing, photographic documentation, and histopathological analysis via H&E staining. The weight and fluorescence intensity of the isolated lung tissues indicated that PTX Emul plus CHA-SME had the lowest lung tissue weight and fluorescence intensity, confirming the above results (Fig. 12C, G, and H). Furthermore, the metastatic tumor nodules distributed in the lung tissue were observed. Similarly, compared with the control group, which exhibited extensive tumor metastasis, the combination of PTX Emul and CHA-SME markedly reduced the number of metastatic nodules in the lungs (Fig. 12I). In addition, in vivo toxicity was preliminarily evaluated by monitoring changes in mouse body weight (Fig. 12F). While body weight remained stable in all treatment groups relative to the control, a decrease was observed in the high-dose PTX Emul group (45 mg/kg). Therefore, the PTX Emul plus CHA-SME could effectively inhibit lung metastasis of 4T1 breast tumors through stimulated systemic immune responses.

## Conclusion

In this study, we developed a coordinated immunochemotherapy strategy by combining TNBC-targeted PTX Emul with lymph-node-targeted CHA-SME. This dual-pronged strategy was designed to elicit potent ICD within the tumor microenvironment and to simultaneously activate immune cells in the lymph nodes, thereby achieving effective immunotherapy against TNBC. The TNBC-directed PTX Emul markedly enhanced tumor accumulation of PTX and efficiently induced ICD, as demonstrated by pronounced CRT exposure and increased release of HMGB1 and ATP. Concurrently, CHA-SME promoted lymphatic transport and selective accumulation of CHA in the MLNs, leading to activation of DCs and T cells. Consequently, the combination of PTX Emul and CHA-SME markedly enhanced the antitumor immune response through 3 interconnected mechanisms: robust ICD induction in the orthotopic 4T1 tumor microenvironment, potent DC maturation, and effective activation of T cell-mediated immunity. This integrated strategy resulted in significantly inhibited growth of orthotopic 4T1 tumors and, notably, reduced lung metastasis. Together, this dual-pronged immunochemotherapy platform presents a promising strategy for potent and coordinated treatment of TNBC.

Looking forward, the promising preclinical efficacy and mechanistic insights presented here support further development of this dual-pronged platform. Subsequent steps will include comprehensive safety and pharmacokinetic studies in higher species, formulation optimization for scalable production, and, ultimately, the design of early-phase clinical trials to evaluate the safety and preliminary efficacy of this combination strategy in patients with advanced or metastatic TNBC.

## Materials and Methods

### Preparation and characterization of PTX Emul and CHA-SME

The preparation methods and physicochemical characterization of PTX Emul and CHA-SME are detailed in the Supplementary Materials.

### In vitro cellular uptake profile of PTX Emul

4T1 cells were counted using a Countstar automated cell counter (Countstar Mira FL, Shanghai, China) and seeded overnight at 37 °C in either 12-well plates (2 × 10^5^ cells/well) or confocal dishes (1 × 10^5^ cells/dish). In the immunofluorescence assay, 4T1 cells were exposed to free Cou-6 or Cou-6-labeled PTX Emul under identical conditions (Cou-6: 100 ng/ml) for specified time intervals (1, 2, 4, and 6 h). Following 3 PBS washes, cells were mounted in DAPI-containing antifade medium and imaged using a Cytation 5 system (Biotek). For the CLSM assay, 4T1 cells in confocal dishes were exposed to free Cou-6 or Cou-6-labeled PTX Emul for 2 h, washed, and fixed as above. The cellular cytoskeleton was stained with TRITC phalloidin (Yeasen, Shanghai, China) for 30 min. The cells underwent 2 washes using PBS before mounting in an antifade mounting medium with DAPI and then imaged on CLSM (TCS SP8X). Furthermore, 4T1 cells were incubated with an excess of DiI-LDL (50 and 100 μg) for 1 h and then cultured in Cou-6-labeled PTX Emul to verify whether PTX Emul could be uptaken by 4T1 cells through the LDL receptor-mediated cellular uptake mechanism. CLSM assay was observed using the above methods. For flow cytometry analysis, cells were processed post-incubation by washing 3 times with PBS, followed by centrifugation to harvest the cell pellets for analysis.

### 3D 4T1-tumor spheroids’ penetration capability

The spheroids’ penetration capability of PTX Emul was evaluated by a 3D 4T1-tumor spheroid formation assay. Briefly, 4T1 cell spheroids were initiated by seeding 1 × 10^3^ cells/well in a round-bottom 96-well plate (ultralow attachment), followed by a brief centrifugation (1,000 × *g*, 10 min). After 5 days, 4T1-tumor spheroids were incubated with Cou-6 or Cou-6-labeled PTX Emul at the Cou-6 concentration of 100, 200, and 500 ng/ml, respectively. Fluorescent images of the tumor spheroids were acquired utilizing the Cytation 5 at various incubation intervals, specifically at 1, 2, 4, 6, and 8 h. For the CLSM assay, 4T1-tumor spheroids were incubated with Cou-6 or Cou-6-labeled PTX Emul for 4 and 8 h. Subsequently, the cytoskeleton and nucleus were stained with TRITC phalloidin and DAPI, respectively, and the specimens were analyzed using CLSM.

### In vitro cytotoxicity assay

The cytotoxic effect of PTX Emul on 4T1 cells was assessed via the Cell Counting Kit-8 assay (Dojindo Laboratories). The cells were treated with increasing concentrations of PTX Emul at different concentrations. Cell viability was determined following the manufacturer’s standard protocol. The apoptosis-related protein expression was detected by Western blot. Following preparation using the aforementioned methods, the 3D 4T1-tumor spheroids were incubated in a medium supplemented with PTX Emul at specified concentrations. The size and area of spheroids were observed and calculated by the Cytation 5 every 2 days. Following 2 PBS washes, tumor spheroids were stained with Live/Dead Cell Double Staining Kit (Abbkine) and then imaged to estimate viability.

### ICD-inducing ability and DC maturation

The key hallmarks of ICD—intracellular HMGB1 release and cell surface CRT exposure—were evaluated. HMGB1 and CRT expression were tested by CLSM and flow cytometry analysis. In short, 4T1 cells were exposed to varying concentrations of PTX Emul for 24 h. Subsequently, the cells underwent incubation with target-specific antibodies: for HMGB1, a primary antibody (ab18256, Abcam) followed by an Alexa Fluor 647 secondary (ab150083, Abcam); for CRT, a direct Alexa Fluor 488 conjugate (#62304S, Cell Signaling Technology). Protein localization was assessed by CLSM. Surface CRT expression levels were quantified in parallel by flow cytometry on identically stained samples. In addition, the ICD effects, including HMGB1 and CRT, induced by PTX Emul on 3D 4T1-tumor spheroids were also investigated using the methods described above.

Bone marrow-derived DCs (BMDCs) were extracted from the bone marrow of BALB/c mice and subsequently cultured to promote their differentiation. To study their maturation, these immature BMDCs (5 × 10^5^/well) were then co-cultured with 4T1 cells (1 × 10^5^/well). Prior to co-culture, the 4T1 cells had been treated for 24 h with either PBS or PTX Emul (0.1, 0.3, and 0.5 μg/ml) and washed. After 24 h of co-incubation, cells were collected, stained for surface markers (CD45, CD11c, CD80, CD86, and MHC-II), and analyzed by flow cytometry to evaluate BMDC maturation.

### In vivo biodistribution of PTX Emul and CHA-SME

The in vivo tissue biodistribution of PTX Emul, administered through tail vein injection, and CHA-SME, administered orally, was assessed in orthotopic 4T1 tumor-bearing BALB/c mice. Detailed descriptions of the specific experimental groups and methodologies are provided in the Supplementary Materials.

### In vivo antitumor and anti-metastatic efficacy

For the evaluation of in vivo antitumor efficacy, orthotopic 4T1 tumor-bearing BALB/c mice were randomized into 5 groups (*n* = 7 per group): Control, PTX Emul (15 mg/kg, i.v.), PTX Emul (45 mg/kg, i.v.), CHA-SME (35 mg/kg, p.o.), or PTX Emul (15 mg/kg, i.v.) plus CHA-SME (35 mg/kg, p.o.). CHA-SME was administered orally once daily, and PTX Emul was administered intravenously once every 3 days. During the administration period, mice were weighed daily, and tumor size was measured regularly at 2-day intervals, with the volume being calculated using the following formula: Tumor volume = [(Length) × (Width)^2^] × 0.52 [50]. On day 15, peripheral blood was collected, and the mice were euthanized by cervical dislocation. Tumors were carefully excised, weighed, and photographed. Immune cells present within tumor tissues were analyzed utilizing flow cytometry, with the specific methodologies comprehensively detailed in the Supplementary Materials. The safety of the combination treatment was evaluated by examining pathological changes in major tissue organs, as well as measuring blood biochemistry and routine blood parameters.

For the evaluation of in vivo antitumor metastasis, a lung metastatic TNBC model was employed. Detailed experimental protocols are provided in the Supplementary Materials.

### Statistical analysis

Data were analyzed with GraphPad Prism software (version 7.00, GraphPad Software, La Jolla, CA, USA). For comparisons involving 2 groups, an unpaired Student’s *t* test was applied; for comparisons across more than 2 groups, one-way analysis of variance (ANOVA) was used. Statistical significance was defined as *P* < 0.05.

## Data Availability

The data supporting the results of this study can be obtained from the corresponding authors upon reasonable request.
