# Supplementary material for: Dual-Pronged Lipid Nanocarriers Promote Immunotherapy for TNBC by Inducing Immunogenic Cell Death and Activating Lymphoid Immune Cells
Source: Research (Wash D C). 2026 Apr 13;9:1139. doi: 10.34133/research.1139 (PMC13075195; doi:10.34133/research.1139)
Supplement: Supplementary 1 — Materials and Methods Figs. S1 to S8 [file research.1139.f1.docx]

**Supplementary Materials**

**Materials and Methods**

**Materials**

PTX was procured from Guilin Huiang Biopharmaceutical (Guilin, Guangxi, China). CHA was generously supplied by Sichuan Jiuzhang Biotech (Chengdu, Sichuan, China). Coumarin-6 (Cou-6) was obtained from Sigma-Aldrich (Saint Louis, MO, USA). DiR was sourced from Beijing LABLEAD Inc. (Beijing, China). DAPI was procured from Beijing Solarbio Science & Technology Co., Ltd (Cat C0066; Beijing, China).

**Cell lines and cell culture**

The 4T1 and 4T1-luc cell lines were obtained from the Cell Culture Center of the Institute of Basic Medical Sciences, Peking Union Medical College (Beijing, China) and Shanghai Sciencelight Biology Science & Technology (Shanghai, China), respectively. Both cell lines were cultured in high-glucose DMEM with 10% fetal bovine serum (FBS; Sino Biological, Beijing, China).

**Animals**

Female BALB/c mice (18–20 g) were sourced from Beijing Vital River Laboratory Animal Technology (Beijing, China). All animal procedures performed in this study were conducted in accordance with the approved guidelines and were reviewed and approved by the Animal Care and Welfare Committee of the Institute of Materia Medical, Chinese Academy of Medical Sciences & Peking Union Medical College (Approval Nos. 00008679 and 00004423).

**Preparation and characterization of PTX Emul and CHA-SME**

For the preparation of PTX Emul (100 mL), an oil phase containing soybean oil (10.0 g), medium-chain triglycerides (10.0 g), and vitamin E acetate (10.0 mg) was homogenized by stirring at 60 °C. The PTX-cholesterol complex (170.9 mg) was dissolved in the oil phase at the same temperature. Separately, an aqueous phase was prepared by dispersing soybean lecithin (1.2 g), poloxamer 188 (2.0 g), and glycerol (2.25 g) in ultrapure water (73.0 mL) at 60 °C. This aqueous phase was gradually combined with the oil phase at 60 °C and subjected to high-speed shearing at 19,000 rpm for 8 min. The resulting coarse emulsion was further processed by high-pressure homogenization at 800 bar for eight cycles to obtain the final emulsion. The pH of PTX Emul was adjusted to 4.5 using 0.1 M hydrochloric acid. After purging with nitrogen gas, the emulsion was aseptically aliquoted into 10 mL portions and sterilized by autoclaving at 115 °C for 30 min.

CHA and phospholipids at a mass ratio of 1:2.2 were dissolved in absolute ethanol and stirred for 15 min at room temperature. Subsequently, the organic solvent was evaporated under reduced pressure using a rotary evaporator. The resulting CHA-phospholipid complex was collected, dried under vacuum, weighed, and stored at –20°C until further use. For the preparation of the CHA-phospholipid complex-loaded self-microemulsifying drug delivery system (CHA-SMEDDS), an accurately weighed quantity of the CHA-phospholipid complex was mixed with a homogeneous solution composed of ethyl oleate, Labrasol, and Transcutol HP in a mass ratio of 1:3:2. The mixture was then vigorously shaken at room temperature until a transparent solution was obtained.

Following preparation, the average particle size, size distribution, and zeta potential of PTX Emul and CHA-SME were determined using dynamic light scattering (DLS). The morphological characteristics of PTX Emul and CHA-SME were observed via transmission electron microscopy (TEM).

**In vivo biodistribution of PTX Emul and CHA-SME**

4T1 cells were inoculated into the right mammary fat pad of BALB/c mice to obtain an orthotopic 4T1 breast tumor. The in vivo tissue biodistribution of PTX Emul was evaluated in orthotopic 4T1 tumor-bearing BALB/c mice following tail vein injection. Mice with a tumor burden of 300–500 mm^3^ were administered free DiR or DiR-labeled PTX Emul at a dose of 1 mg/kg DiR via tail vein injection (n = 4 per group). In vivo fluorescence imaging was performed at predetermined time points post-injection to assess the in vivo biodistribution of DiR-labeled PTX Emul. Free DiR served as the control. Image data from the in vivo fluorescence imaging were used in semi-quantitative calculations to further evaluate the tumor-targeting ability of PTX Emul.

The in vivo biodistribution of CHA-SME was evaluated following oral administration in orthotopic 4T1 tumor model mice (tumor volume 300-500 mm^3^), which were randomized to receive free DiR or DiR-labeled SME (1 mg DiR/kg) by gavage (n=3/group). Two hours after administration, the mice were sacrificed, and the small intestine and MLNs were carefully isolated and subjected to ex vivo fluorescence imaging. Free DiR served as the control. Image data from the ex vivo fluorescence imaging were used in semi-quantitative calculations to further evaluate the MLNs-targeting ability of CHA-SME.

**Analysis of immune cells within tumor tissue**

Tumors were cut into small pieces and digested with collagenase IV (Gibco) and DNase I (Sigma) to a single-cell suspension. Tumor cells were washed twice and resuspended in PBS with Zombie Fixable Viability dye. Then, the cells were washed, and Brilliant Staining Buffer (BD Biosciences) and Fc block were added. After 15 min, cells were stained with FITC anti-CD45 antibody, APC/Cyanine anti-TCRβ antibody, PerCP/Cyanine5.5 anti-CD4 antibody, PE/Cyanine7 anti-CD8 antibody. All the antibodies used in flow cytometry were from BioLegend. Data were collected by a BD FACSCelesta™ Multicolor Flow Cytometer (BD Biosciences) and analyzed by FlowJo software.

**In vivo anti-tumor metastasis efficacy**

A lung metastatic TNBC model was established via intravenous injection of 4T1-Luc cells (5×10^5^ cells per mouse). Four days post-inoculation (designated Day 0), mice were randomized into five groups (n = 3) for treatment: Control, PTX Emul (15 mg/kg, i.v.), PTX Emul (45 mg/kg, i.v.), CHA-SME (35 mg/kg, p.o.), or PTX Emul (15 mg/kg, i.v.) combined with CHA-SME (35 mg/kg, p.o.). Metastatic progression was monitored by In Vivo bioluminescence imaging (IVIS Spectrum, PerkinElmer) on days 14 and 16. At the experimental endpoint, lungs were harvested for ex vivo fluorescence imaging and histological analysis.


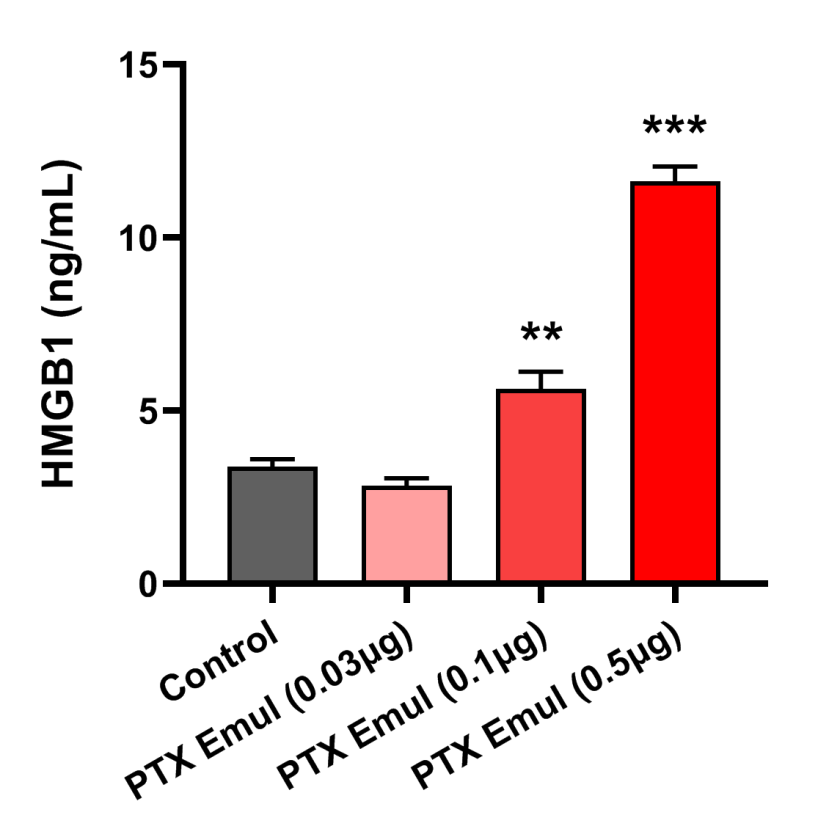
**
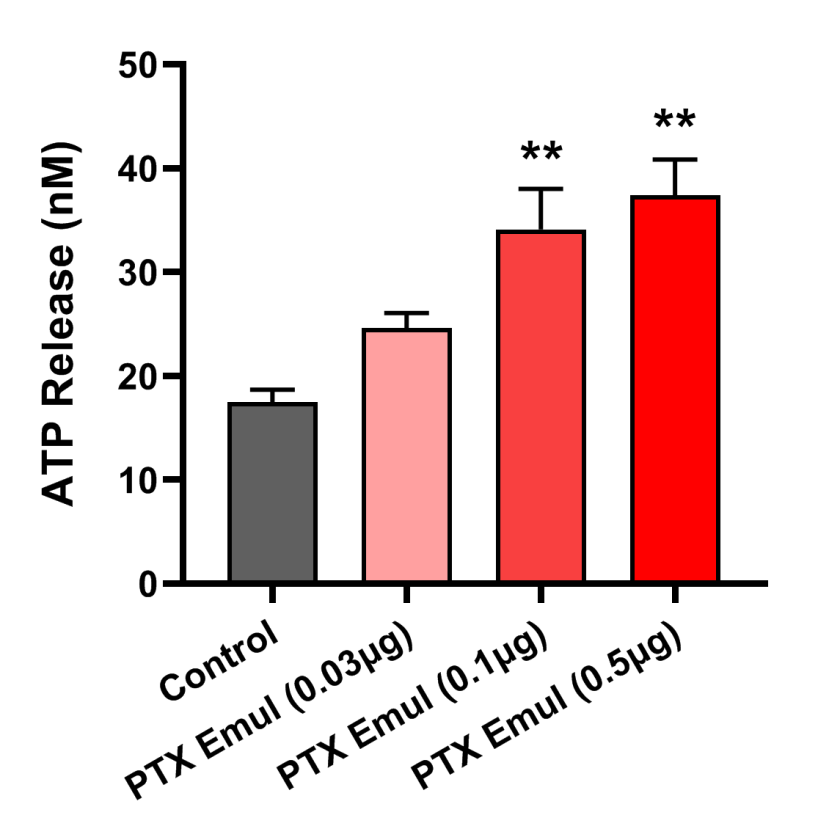
Fig. S1.** ATP levels secreted by 4T1 cells after incubation with PTX Emul. Each value represents the mean ± SEM (n = 3). **p < 0.01 compared with the control group.

**Fig. S2.** HMGB1 levels released by 4T1 cells after incubation with PTX Emul. Each value represents the mean ± SEM (n = 3). **p < 0.01 and ***p < 0.001 compared with the control group.


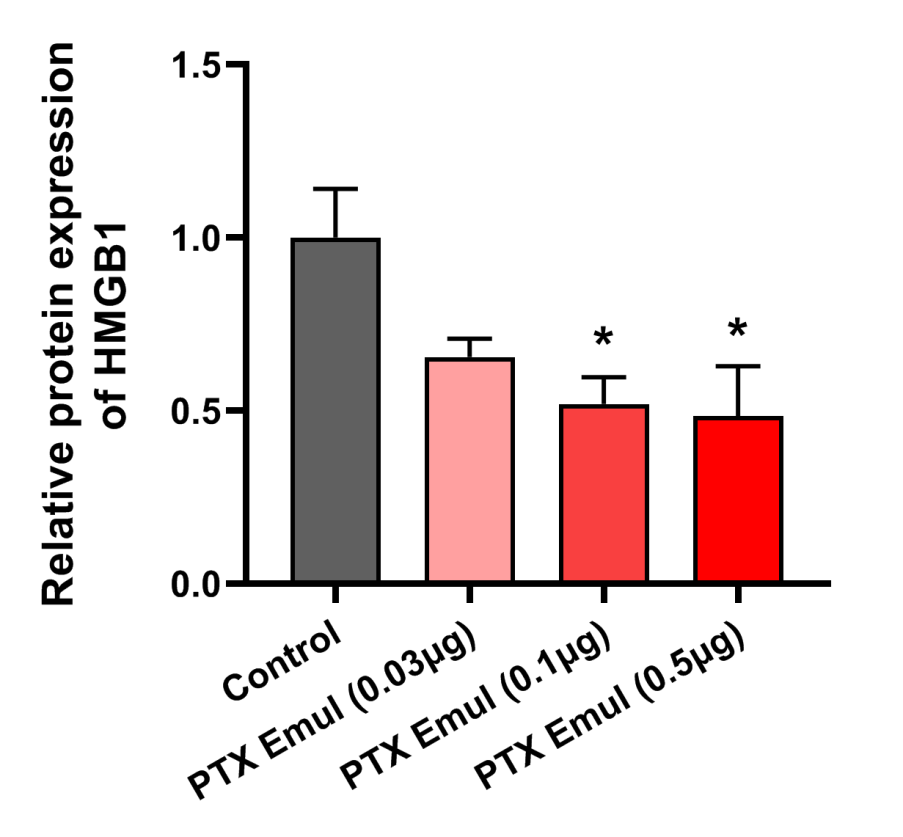

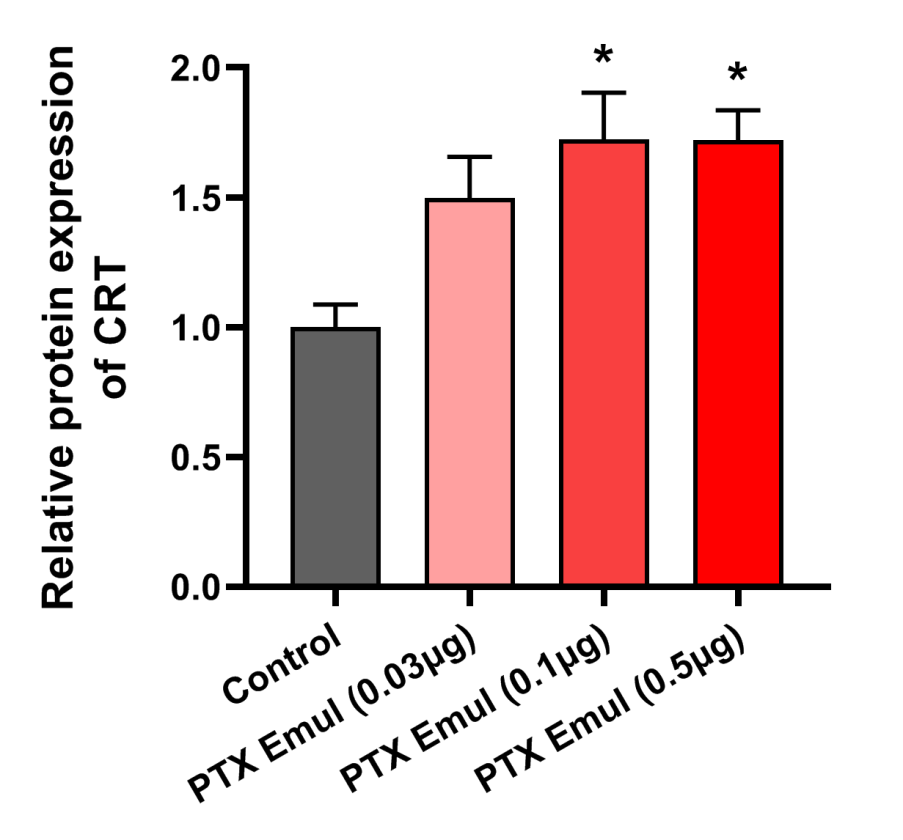
**Fig. S3.** Relative protein expression of CRT in 4T1 cells after incubation with PTX Emul for 24 h. Each value represents the mean ± SEM (n = 3). *p < 0.05 compared with the control group.

**Fig. S4.** Relative protein expression of HMGB1 in 4T1 cells after incubation with PTX Emul for 24 h. Each value represents the mean ± SEM (n = 3). *p < 0.05 compared with the control group.

**
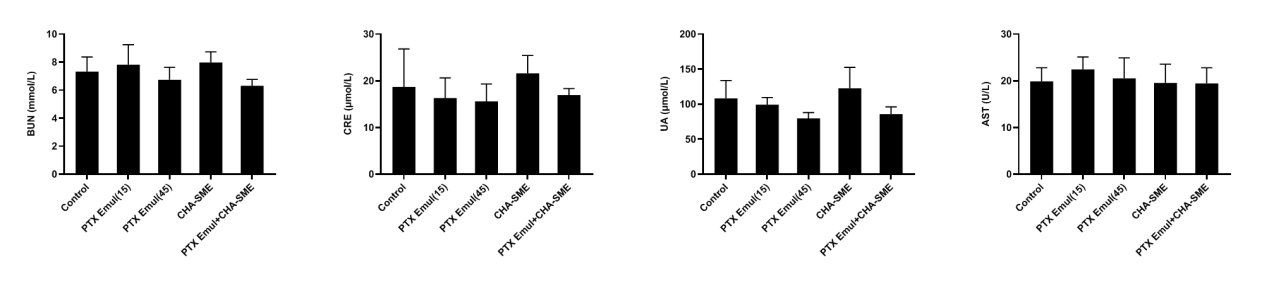
Fig. S5.** The determination of blood biochemistry parameters, including BUN, CRE, UA, and AST levels. Each value represents the mean ± SD (n = 7).


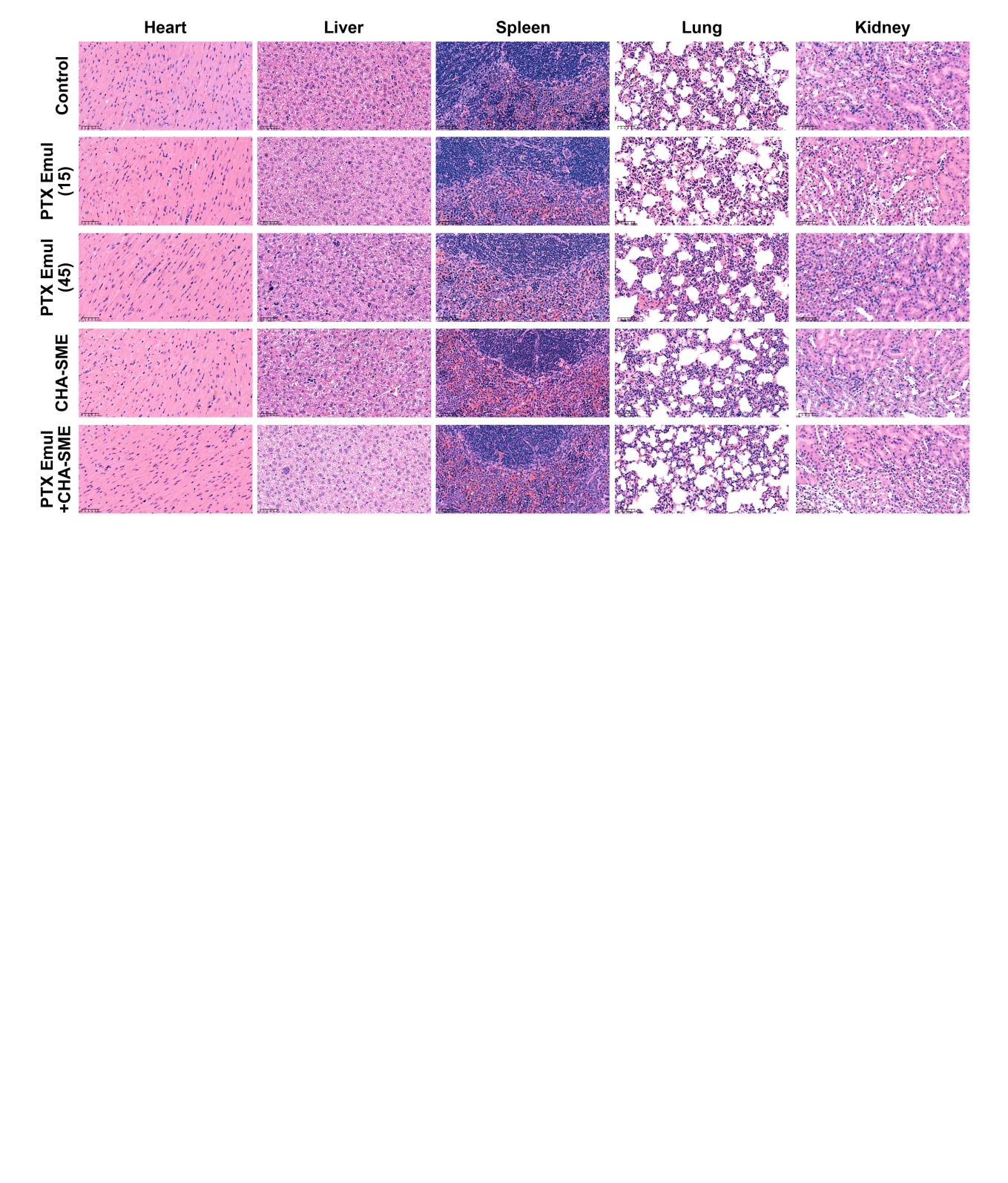


**Fig. S6.** Histopathologic analyses of H&E-stained tissue sections from heart, liver, spleen, lung, and kidney after the indicated treatment. Scale bar = 50 µm.

**Fig. S7.** Multiplex immunofluorescent staining of CD8+T cells infiltrated in the tumors after different treatments. Scale bar = 100 μm.
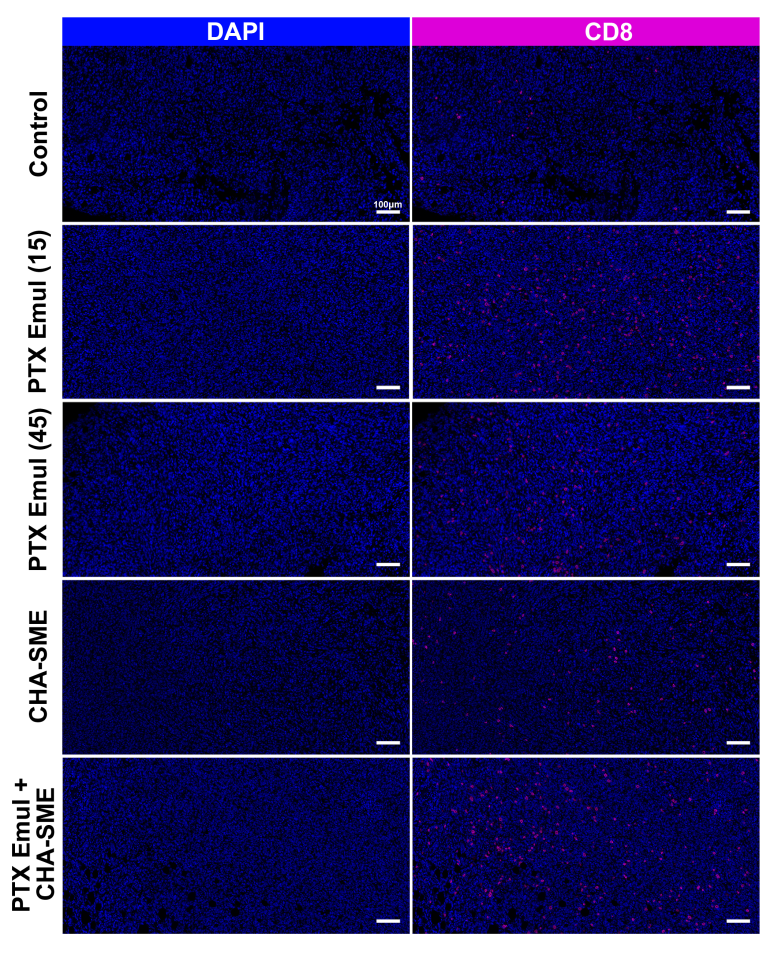


**Fig. S8.** Cytokine secretion of IL-1α, TNF-α, and IFN-β in the plasma after different treatments.
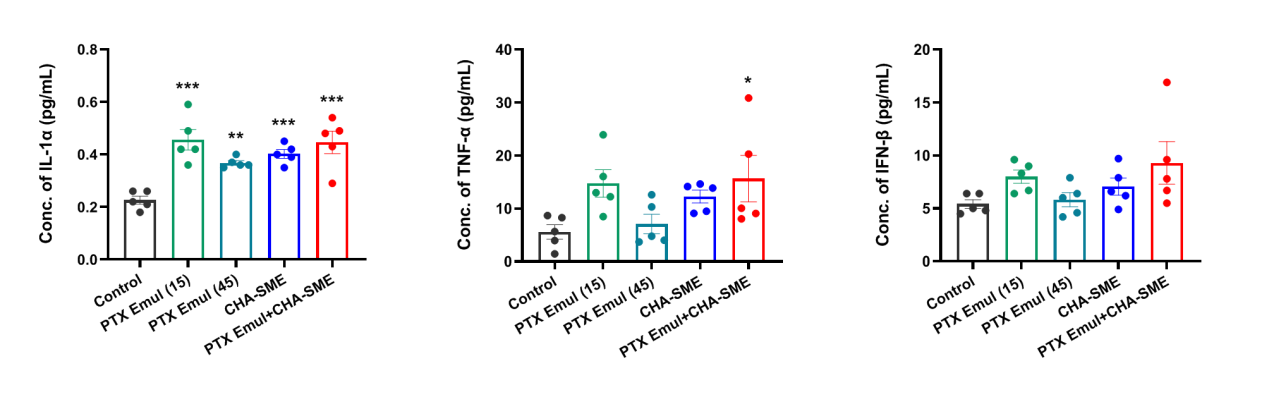
 Each value represents the mean ± SEM (n = 5). *p < 0.05, **p < 0.01, and ***p < 0.001 compared with the control group.
